# Oxidative Stress in Tauopathies: From Cause to Therapy

**DOI:** 10.3390/antiox11081421

**Published:** 2022-07-22

**Authors:** Fernando Bartolome, Eva Carro, Carolina Alquezar

**Affiliations:** 1 Group of Neurodegenerative Diseases, Hospital Universitario 12 de Octubre Research Institute (imas12), 28041 Madrid, Spain; fbartolome.imas12@h12o.es; 2 Network Center for Biomedical Research in Neurodegenerative Diseases (CIBERNED), Spain; eva.carro@isciii.es; 3 Neurobiology of Alzheimer’s Disease Unit, Chronic Disease Program, Instituto de Salud Carlos III, 28222 Madrid, Spain

**Keywords:** oxidative stress, antioxidants, tau, tauopathies

## Abstract

Oxidative stress (OS) is the result of an imbalance between the production of reactive oxygen species (ROS) and the antioxidant capacity of cells. Due to its high oxygen demand, the human brain is highly susceptible to OS and, thus, it is not a surprise that OS has emerged as an essential component of the pathophysiology of several neurodegenerative diseases, including tauopathies. Tauopathies are a heterogeneous group of age-related neurodegenerative disorders characterized by the deposition of abnormal tau protein in the affected neurons. With the worldwide population aging, the prevalence of tauopathies is increasing, but effective therapies have not yet been developed. Since OS seems to play a key role in tauopathies, it has been proposed that the use of antioxidants might be beneficial for tau-related neurodegenerative diseases. Although antioxidant therapies looked promising in preclinical studies performed in cellular and animal models, the antioxidant clinical trials performed in tauopathy patients have been disappointing. To develop effective antioxidant therapies, the molecular mechanisms underlying OS in tauopathies should be completely understood. Here, we review the link between OS and tauopathies, emphasizing the causes of OS in these diseases and the role of OS in tau pathogenesis. We also summarize the antioxidant therapies proposed as a potential treatment for tauopathies and discuss why they have not been completely translated to clinical trials. This review aims to provide an integrated perspective of the role of OS and antioxidant therapies in tauopathies. In doing so, we hope to enable a more comprehensive understanding of OS in tauopathies that will positively impact future studies.

## 1. Introduction: Oxidative Stress and Tauopathies

Oxidative stress (OS) is defined as the imbalance between pro-oxidants and antioxidants. The most common pro-oxidants are reactive oxygen species (ROS), which are chemically reactive molecules containing oxygen. These molecules result from normal cell metabolism and include superoxide anion (O^−2^), hydroperoxyl radical (HO_2_), hydrogen peroxide (H_2_O_2_), and hydroxyl radical (OH), among others [1]. In mammalian cells, the major source of ROS is mitochondria, where ROS are produced permanently as a byproduct of ATP production by the electron transport chain [2]. ROS regulate important biological processes such as cell proliferation, host defense and gene expression [3,4] and, thus, normal ROS levels are essential to maintain the correct function of the organism. On the other hand, elevated levels of ROS are highly toxic as they damage essential macromolecules, such as DNA, RNA, proteins and lipids [5]. In order to manage ROS, cells synthesize molecules that display anti-oxidant properties, which include antioxidant enzymes such as the cytosolic Cu/Zn-superoxide dismutase or superoxide dismutase 1 (Cu/Zn-SOD, SOD1), the mitochondrial manganese superoxide dismutase or superoxide dismutase 2 (Mn-SOD, SOD2), catalase (CAT), glutathione peroxidase (GPx), and glutathione reductase (GR) [6] and non-enzymatic antioxidants such as metal binding proteins (MBPs), glutathione (GSH), uric acid (UA), melatonin (MEL), bilirubin (BIL) and polyamines (PAs) [7].

**Figure 1:**
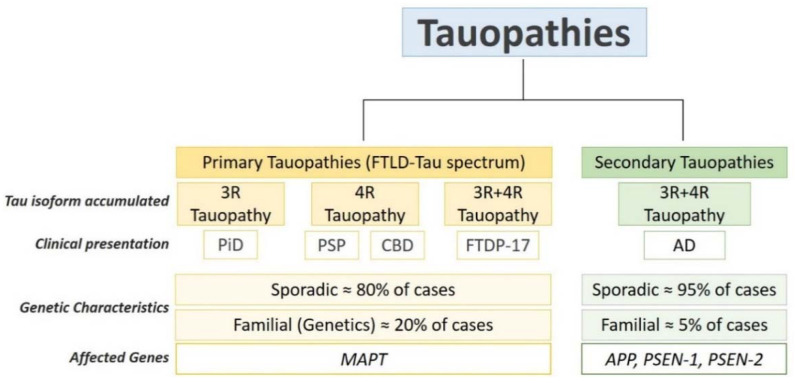
Pathologic and genetic characteristics of tauopathies. Tauopathies are complex diseases regarding pathology, clinical presentation and genetics. Pathologically, tauopathies are classified in primary and secondary tauopathies. Primary tauopathies fall under the umbrella of frontotemporal lobar degeneration with tau inclusions (FTLD-Tau) and include Pick’s disease (PiD), progressive supranuclear palsy (PSP), corticobasal degeneration (CBD), and frontotemporal dementia with Parkinsonism (FTDP-17). Alzheimer’s disease in a secondary tauopathy characterized by the presence of extracellular inclusions containing Amyloid-β (Aβ) protein. Depending on tau isoform composing the intracellular inclusions, tauopathies are classified in 3R-tauopathies (PiD), 4R tauopathies (PSP and CBD) and 3R:4R tauopathies (FTDP-17 and AD). The majority of tauopathy cases are sporadic. Mutations in *MAPT* account of around 20% of the FTLD-tau cases. Mutations in amyloid-*beta precursor protein (APP) Presenilin-1 and Presenilin-2 (PSEN-1,2) genes account* for around 5% of AD cases.

Elevated OS is considered an essential component of the pathophysiology of several syndromes including different types of cancer and neurodegenerative diseases such as tauopathies [8]. Tauopathies are a clinically, pathologically, biochemically, and morphologically heterogeneous group of age-related neurodegenerative disorders, characterized by the presence of cytosolic aggregates containing aberrant forms of the microtubule-associated protein tau [9]. The most common tauopathies are Alzheimer’s disease (AD) and frontotemporal lobar degeneration Tau (FTLD-Tau), which encompass a spectrum of several syndromes such as progressive supranuclear palsy (PSP), corticobasal degeneration (CBD), Pick’s disease (PiD), and frontotemporal dementia with Parkinsonism linked to chromosome 17 (FTDP-17). AD is a secondary tauopathy because patients present extraneuronal deposits of amyloid-beta (Aβ) protein, in addition to the intraneuronal tau inclusions [10]. The rest of the tauopathies are considered primary tauopathies because tau protein is the only component of the pathological deposits [11]. Previous reviews have extensively discussed the neuropathology, symptomatology and genetics of tauopathies, and can be consulted to widen knowledge of these syndromes [9,12,13]. A summary of the main characteristics of tauopathies is shown in Figure 1.

The human brain is highly susceptible to OS [14]. Neurons require large amounts of energy and high oxygen supply [15], entailing an elevated rate of ROS production in the human brain. However, neuronal cells exhibit low levels of antioxidants [16] and, thus, these cells are very susceptible to ROS accumulation [17,18]. Elevated ROS levels lead to OS, which promotes the destruction of cellular components and ultimately cell death via apoptosis or necrosis (6), suggesting that OS might be responsible of neuronal death in tauopathies. Indeed, the neurons of tauopathy patients and animal models display elevated OS For example, the Pick bodies of PiD patients and the threads and glial inclusions of CBD patients exhibited increased levels of the oxidative marker heme oxygenase-1 (HO-1) [19]. Furthermore, elevated OS and increased levels of the lipid peroxidation marker 4-hydroxynonenal (4-HNE) were found in a mouse model of FTLD-tau (P301S transgenic mice) [20] and in the frontal cortex of FTLD-tau patients carrying the P301L tau mutation [21]. PSP patients also displayed elevated levels of several OS markers such as malondialdehyde (MDA) and thiobarbituric acid reactive substances (TBARS) [22,23,24] and antioxidants such as SOD1, SOD2 and GPx [25,26]. Elevated OS is also closely associated with AD [27] and several publications have extensively reviewed the role of OS in AD [28,29,30,31,32]. Briefly, during the course of AD several manifestations of OS occur including the dysregulation of antioxidant enzymes, the oxidation of proteins, lipids and DNA, the formation of toxic substances such as peroxides, alcohols, aldehydes, free carbonyls, ketones, cholestenone, and the oxidative modifications in nuclear and mitochondrial DNA. AD is a secondary tauopathy characterized by the presence of intraneuronal tau aggregates and extracellular deposition of Aβ plaques. Several studies in patients and transgenic animal models showed that Aβ and OS are closely related because Aβ induces OS, and OS increases Aβ production [33], suggesting that OS might not only be related with tau accumulation but also with other pathologic events that occur in tauopathies.

Nowadays, tauopathies are still incurable and difficult to treat. With the prevalence of these diseases increasing worldwide, it, is highly necessary to identify treatments to cure and/or prevent these devastating diseases. To do so, the molecular mechanisms underlying tauopathies should be fully understood. Since OS is considered a key component of the pathophysiology of tauopathies, in this review, we delve into the role of OS in tauopathies. We have compiled current knowledge about the mechanisms underlying OS in tauopathies and discussed whether OS triggers tau pathology or it is just a mere consequence of aberrant tau accumulation. Furthermore, we have summarized the antioxidant therapies proposed for tauopathies, discussing their potential effect in clinical trials, as well as the pros and cons of their use in tauopathy patients.

## 2. Molecular Mechanisms Leading to Oxidative Stress in Tauopathies: Implication of Mitochondria and Antioxidant Enzymes

Mitochondrial dysfunction leading to excessive ROS production has been reported in tauopathies [34,35,36,37]. The majority of these studies have been carried out in AD and have demonstrated that AD patients display a significant reduction in healthy mitochondria in favor of the increase in damaged mitochondria [38], as well as abnormalities in mitochondrial structure and mitochondrial fission and fusion [39,40]. The impairment of mitochondrial function has also been observed in animal models of AD and other tauopathies. For example, it was reported that mitochondrial function and respiration was notably compromised in two triple transgenic AD mouse models (the ^triple^AD and 3xTg-AD) [41,42]. Furthermore, alterations in mitochondrial dynamics, content and transport have been reported in FTLD-tau mice models such as K3 mice (human K369I mutant tau) [43], rTg4510 mice (repressible human P301L mutant tau) [44] and KI-P301L mice (P301L tau knock-in) [45]. Interestingly, transgenic mice models expressing human P301L mutant tau showed alterations in mitophagy, the selective mechanism of mitochondria degradation [46], which were accompanied by altered mitochondrial bioenergetics [41,47].

Tau overexpression, both wild-type (WT) and mutant, induced the impairment of mitochondrial function in transgenic mice and cellular models [36,41,42,43,44,45,48,49], suggesting that mitochondrial dysfunction might be associated with tau accumulation independently of the presence or absence of mutations. The link between tau protein and mitochondrial function has been largely reported. Tau interacts with mitochondrial proteins and impairs mitochondrial bioenergetics and dynamics [50]. Furthermore, tau influences mitochondrial transport along the neuronal axon [51,52]. Several studies have demonstrated that the abnormal forms of tau protein accumulated in tauopathies impaired mitochondrial function. Pathological tau reduced complex I activity, causing a reduction in ATP generation, mitochondrial membrane potential (ΔΨm) dissipation, promotion of mitochondrial fission and fragmentation and, finally, mitochondrial dysfunction in AD [53]. Studies performed in animal and cell models confirmed that pathological tau also induced mitochondrial dysfunction in primary tauopathies such as FTDL-Tau. For example, P301L mutant tau expressed in transgenic mice induced mitochondrial dysfunction, ROS production, lipid peroxidation, OS and neuronal loss [54,55,56,57]. Furthermore, a proteomic analysis in P301L tau transgenic mice identified the downregulation of proteins involved in mitochondrial respiration and metabolism (mainly components of mitochondrial complex V), leading to mitochondrial dysfunction [47]. The effect of P301L mutant tau disrupting mitochondrial function was recapitulated in SH-SY5Y cells overexpressing P301L tau [58,59]. Other tau mutations related with FTLD-tau also induced mitochondrial dysfunction in induced pluripotent stem cells (iPSC)-derived neurons; however, the mechanism seemed different. The tau protein carrying the 10 + 16 *MAPT* mutation induced mitochondrial dysfunction associated with a hyperpolarization of the mitochondria [36] and TauP301L and TauV337 mutations reduced tau binding to mitochondrial proteins impairing bioenergetics [50]. On the other hand, while the effect of mutant tau in mitochondrial dysfunction is well recognized, the role of WT tau in mitochondrial function is controversial. Some evidence has suggested that WT tau overexpression induced mitochondrial dysfunction in cell cultures [60] but others showed that the overexpression of WT tau improved mitochondrial function [58]. It is important to highlight that different cell types were used in these two papers, suggesting that the accumulation of WT tau might have different effects depending on the cell type. Interestingly, tau −/− mice displayed improved mitochondrial function and reduced oxidative damages [61], suggesting that WT tau levels directly regulate mitochondrial function. Since the majority of tau accumulated in tauopathies is WT tau, additional studies should be carried out to completely understand how exactly WT tau regulates mitochondrial function. In this regard, it has been speculated that pathological forms of WT tau, such as truncated tau [62], might impair mitochondria. In primary neuronal cultures, an overexpressed N-terminal tau fragment (NH_2_-26–44) localized in the mitochondrial membrane and impaired ΔΨm and ATP synthesis [63]. Interestingly, the interaction of this fragment with the mitochondria was favored by Aβ [64]. Supporting this, another report showed that the expression of the Asp421 tau fragment, cleaved by caspase-3, induced mitochondrial failure only in presence of Aβ treatment [65]. This evidence suggests that this specific pathological tau fragment might induce mitochondrial dysfunction in AD, but not in primary tauopathies. Although the evidence presented above suggests that abnormal tau accumulation might be the main inducer of mitochondrial OS in tauopathies, it has been shown that OS might occur in the early stages of the disease, even before the deposition of abnormal tau [47,66]. The results from these studies suggested that mitochondria might not be the major source of ROS in the early progression of the disease; however, the resource of this early OS stress has not yet been described. Since it is well known that OS induces tau pathogenesis (See Section 3 of this review), we speculate that there is a positive loop where the OS generated in the early stages of the disease induces tau pathology and, as a consequence, pathological tau might promote mitochondrial impairment and more OS, leading to neuronal death.

In addition to mitochondrial dysfunction, OS in tauopathies might be the consequence of the age-dependent decrease in antioxidant molecules [67,68,69]. In this regard, the levels and activity of some antioxidant enzymes such as SOD1, SOD2 and catalase were found decreased in human AD brains [70,71]. Interestingly, the activity of the antioxidant GFH-Px was not altered [70]. Furthermore, studies in animal models have shown that antioxidant enzymes have an important role in tau pathology. For example, the reduction in SOD1 and SOD2 led to increased tau pathology in mice [72] and the downregulation of antioxidants such as SOD2 or Thioredoxin reductase (Trxr) enhanced tau-induced neurodegeneration in a drosophila model [73].

The evidence exposed above demonstrates that in tauopathies, OS is the result of mitochondrial and the consequent ROS production or the impairment of the antioxidant system. Although more research should be conducted to confirm this hypothesis, it is also plausible that alterations in both mechanisms contribute to the pathogenesis of tauopathies.

## 3. Oxidative Stress and Tau Pathogenesis: Cause or Consequence?

As we described above, tauopathies are a heterogeneous group of neurodegenerative diseases characterized by the deposition of abnormal tau aggregates in the cytosol of brain cells, mainly in neurons. In each tauopathy, tau self-assembles to form aggregates with a disease-specific morphology and composition [56,74,75,76,77]. There is a correlation between the tau pathology observed in each tauopathy and the clinical dementia observed in patients (Figure 1). However, it is not yet well understood if the different composition of tau aggregates (3R:4R ratio) is directly related with the pathophysiology of each tauopathy. Understanding the molecular mechanisms underlying the formation of tau aggregates is essential to unravel the etiology of these diseases and discover effective treatments. It has been speculated that OS plays an important role in tau pathogenesis. However, the exact role of OS in tauopathies is controversial. While a large number of studies suggest that OS is an early factor that triggers the pathophysiological processes leading to tauopathy, other evidence show that OS is merely a consequence of pathological tau accumulation and aggregation. Below we summarize the evidence supporting both hypotheses.

### 3.1. Tau Pathogenesis as a Consequence of Oxidative Stress

Many lines of evidence suggest that OS appears before tau aggregation and, thus, is an early event in tau pathogenesis [35,66,73,78,79,80,81]. Tau pathogenesis is defined as the biochemical and molecular mechanisms leading to tau aggregation. It has been demonstrated that post-translational modifications (PTMs) in tau protein contribute to its pathogenesis [82]. PTMs involve the addition of chemical groups, sugars, or proteins to specific residues of the targeted protein. The accumulation of ROS has been directly related with the PTMs of several proteins [83,84], suggesting that both tau PTMs and tau pathogenesis could be a consequence of elevated OS.

Tau is modified by several PTMs including phosphorylation, acetylation, ubiquitination, glycation, glycosylation, SUMOylation, methylation, oxidation, and nitration (for a complete review of tau PTMs, see [82]. Among all tau PTMs, phosphorylation is the most studied. Tau protein can be phosphorylated at multiple sites [82] and it has been demonstrated that phosphorylation regulates tau normal function and pathogenesis [85]. Interestingly, the effects of phosphorylation on tau normal physiology and pathogenesis seem to be site-dependent [82]. Aberrant tau phosphorylation at specific sites impaired its binding to microtubules and promotes tau self-aggregation [86,87,88]. Several evidence demonstrated that OS played a critical role in the induction of aberrant tau phosphorylation, which induced the impairment of its physiological function and pathogenesis [72,78,80,89,90,91]. Several studies showed that OS regulated the activity of tau protein kinases and phosphatases [92,93,94,95,96]. Various studies performed in cell cultures indicated that the activity of the tau kinase glycogen synthase kinase 3 beta (GSK-3β) was upregulated under OS. For example, in HEK293 cells overexpressing human tau, treatment with H_2_O_2_ increased GSK-3β activity and tau hyperphosphorylation at Ser396, Ser404, and Thr231 [97]. Similar results were found in primary cortical neuronal cultures [96,98]. Additionally, the treatment with low concentrations of a GSK-3β inhibitor protected against OS in other cellular cultures [99]. OS also stimulated the activity of GSK-3β, and increased tau phosphorylation in C57BL/6 mice [100]. Besides GSK-3β, OS also affected the activity of other kinases involved in tau phosphorylation, including several tau kinases from the stress-activated protein kinases family such as c-Jun N-terminal Kinase (JNK) and p38 [92,93,94]. It was demonstrated that OS caused the activation of p38, leading to tau hyperphosphorylation in vivo [101]. Furthermore, in differentiated M17 neuroblastoma cells, prolonged OS induction increased tau phosphorylation at Ser396/Ser404, due to increased activity of JNK and p38 [90]. Other studies also linked the elevated OS with the dysregulation of tau phosphatases. In this regard, OS reduced the activity of the protein phosphatase 2 (PP2A) in differentiated M17 neuroblastoma cells, contributing to increased tau phosphorylation and pathogenesis [90]. Additionally, in neuronal cultures, ROS inhibited PP2A and PP5 phosphatases, leading to aberrant tau phosphorylation and neuronal apoptosis [102]. Furthermore, the OS induced by okadaic acid inhibited PP1 and PP2A activity in rat cortical neurons [103] and HT22 cell cultures [104], leading to increased tau phosphorylation and aggregation. Interestingly, another report showed that the induction of OS using H_2_O_2_ resulted in PP1 activation and tau dephosphorylation at specific sites in rat hippocampal cells and SH-SY5Y human neuroblastoma cells [105], suggesting that OS might regulate the phosphorylation/dephosphorylation of tau by the activation/inhibition of phosphatases. In addition to the PPA family, OS also inhibited other tau phosphatases such as calcineurin. It was demonstrated that OS upregulated the regulator of calcineurin 1 (*RCAN1*) gene, inducing calcineurin activity inhibition and, thus, reducing tau dephosphorylation [98]. Although it has been fully demonstrated that OS regulates tau kinases and phosphatases, the exact mechanistic pathways by which OS regulates the activity of these enzymes is not yet understood.

In addition to regulate the activity of tau kinases and phosphatases, OS may also regulate tau pathogenesis by other mechanisms such as the oxidation of fatty acids and lipid peroxidation. Several reports demonstrated that OS induced the oxidation of fatty acids, which seemed to stimulate tau polymerization and consequent hyperphosphorylation [89,106]. Conditioned media from astrocytes treated with saturated fatty acids increased tau phosphorylation at AD-specific sites and antioxidant treatment reduced fatty acids-mediated tau phosphorylation, suggesting that fatty acid oxidation significantly contributed to tau pathogenesis [89]. Lipid peroxidation is also implicated in tau pathogenesis. Lipid peroxidation products such as 4-hydroxy-2-nonenal (HNE) and acrolein increased the assembling capacity of phosphorylated 4R-tau [107], promoted conformational changes and the polymerization tau [108] and promoted tau hyperphosphorylation [109]. Furthermore, another possible link between OS and tau pathogenesis is the peptidyl prolyl *cis*-trans isomerase 1 (PPIase1) or Pin1. This enzyme binds to proteins and isomerizes phospho-Serine/Threonine-Proline motifs and, thus, plays an important role regulating protein phosphorylation. Pin1 was significantly oxidized and downregulated in the brain of AD patients [110]. Furthermore, it was demonstrated that Pin1 was implicated in the dephosphorylation of tau protein [111,112]. Therefore, it is believed that oxidative modifications of Pin1 reduced its activity and promoted tau hyperphosphorylation in AD [113]. Insulin may also play a role in OS-induced tau phosphorylation. It was demonstrated that OS decreased insulin secretion and sensitivity [114] and that insulin regulated the activity of tau kinases/phosphatases [115], suggesting that the decrease in insulin levels associated with OS might lead to tau hyperphosphorylation. To conclude, OS might also induce tau pathogenesis through its role promoting mitochondrial dysfunction. In this regard, mitochondrial SOD2 deficient mice showed increased tau hyperphosphorylation along with mitochondrial dysfunction and OS [72], indicating a positive loop between OS, mitochondrial dysfunction and tau pathogenesis.

Tau protein is also susceptible to be directly modified by oxidation. Oxidation is a PTM that occurs mainly in cysteine and methionine residues [116]. Tau protein presents a pair of cysteine residues (C291/C322) that can be oxidized [82]. Interestingly, protein conformation is very sensitive to oxidation-reduction (redox) changes and, thus, tau oxidation could directly induce its aggregation. Indeed, it was demonstrated that the in vitro oxidation of tau at C-322 (present in R3 repeat) promoted tau aggregation into paired helical filaments [117]. Furthermore, oxidation also regulates tau degradation and, thus, accumulation. A recent study demonstrated that the oxidation of tau at C291/C322 was a prerequisite for completing the internalization of tau through a form of specific protein degradation called endosomal microautophagy (e-MI) [118]. Although there are a few studies suggesting that oxidation might be relevant for tau pathogenesis, the exact role of oxidation on tau function and pathogenesis remains unclear.

The evidence described above provides knowledge about the mechanisms implicated in OS-mediated tau pathology, focusing on the link between OS and tau phosphorylation and oxidation. However, tau protein undergoes other PTMs that are essential to regulate its function and structure [82]. To fully understand how OS induces tau accumulation and aggregation in tauopathies, new research focused on understanding how OS modulates other PTMs should be carried out.

### 3.2. Pathological Tau as Oxidative Stress Inductor

Some studies performed on tauopathy cellular models suggested that aberrant tau might induce ROS production and OS. In this regard, a recent report showed that extracellular tau induced ROS production in cortical co-cultures of neurons and astrocytes [119]. Interestingly, only the insoluble tau aggregates were able to elevate ROS levels. Authors also showed that the extracellular tau aggregates led to the activation of NADPH oxidase without decreasing the level of the endogenous antioxidant glutathione, describing a mechanism by which extracellular tau aggregates might induce OS. Microglia and astrocytes are important sources of ROS [120] and activated microglia are spatially correlated with tau pathology [121,122]. Therefore, it has been suggested that tau might play a role inducing microglial OS. Interestingly, tau aggregates in neuronal-astrocytic co-cultures stimulated NADPH oxidase 2 (NOX) activity and ROS production leading to neuronal death [119]; however, further studies are required to completely understand the relationship between tauopathy and microglial OS. It has also been suggested that truncated forms of tau might induce OS. Truncation is a PTMs observed in the pathological tau accumulated in tauopathy patients [123,124,125]. Cultured cortical neurons obtained from a transgenic rat model expressing truncated human tau presented the depolarization of mitochondria and increased ROS production [126]. Similarly, fragmented tau protein induced copper reduction and contributed to OS by the initiation of copper-mediated generation of H_2_O_2_ [127]. These reports suggest that OS could be the consequence, rather than the trigger, of tau pathology. Interestingly, the overexpression of human WT tau not only induced OS but also increased the sensitivity of neurons to oxidants, likely associated with the tau-related depletion of peroxisomes [54,55]. It is known that OS promotes mitochondrial dysfunction and antioxidants deficiency [72,128], leading to the generation of a more severe oxidative environment [129]. Hence, it is plausible that the increased OS associated with the presence of pathological tau could make the neurons more sensitive to oxidative stressors.

**Figure 2:**
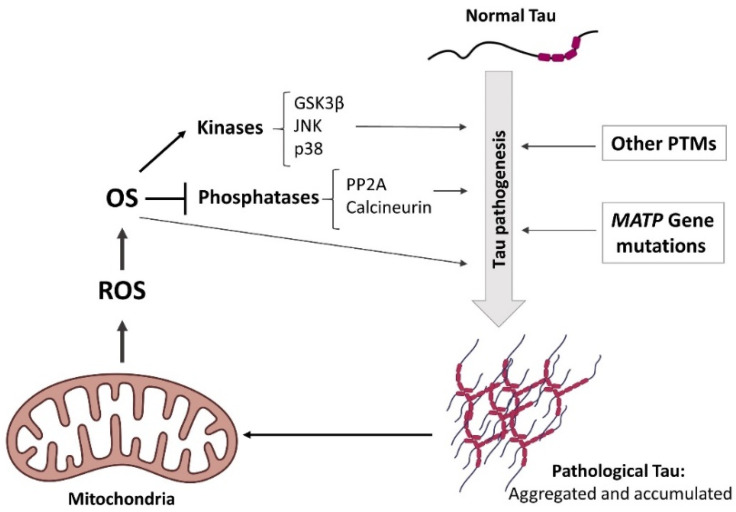
Schematic representation of the positive loop between elevated OS and tau pathogenesis. Mitochondria is the major ROS resource in neurons. Increased ROS leads to elevated OS, which induces tau pathogenesis by the regulation of the activity of tau kinases/phosphatases. On the other hand, the accumulation of pathological forms of tau protein might induce mitochondrial damage and thus, ROS production. We speculate that there is a positive loop where the OS generated in the early stages of the disease induces tau pathology and, in consequence, pathological tau promotes mitochondrial impairment and more OS, leading to neuronal death.

Based in the evidence exposed above, it has been postulated that tau pathology and OS could be two elements of a “vicious circle” in which OS would promote tau pathology and, at the same time, pathological tau accumulation would stimulate ROS production and OS (Figure 2) [79]. The complete understanding of the interplay between oxidative environment and tau pathogenesis may be important to unravel the etiology and tauopathies and for future directions in the development of novel therapeutic options based on antioxidative agents.

## 4. Antioxidant Therapies for the Treatment of Tauopathies

**Figure 3:**
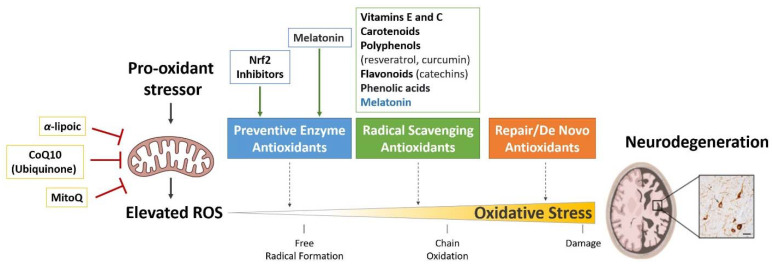
Summary of the antioxidants therapies proposed as good candidates to treat tauopathies. OS is a lineal process that start in the mitochondria by the production of ROS and, if is not prevented, ends with cellular damage and/or cell death. Antioxidants prevent OS by counteracting the damaging action of ROS. Antioxidant enzymes act in the first line of defense suppressing the generation of free radicals. Free radical-scavengers act in the second line of defense interacting with free radicals and neutralizing them to prevent cell damage. The third line of defense consists of restoring the impairment affected by free radicals and they work repairing the damage and reconstituting membranes. Tauopathies antioxidant therapies research has been focused on targeting the first two lines of OS defense by activating antioxidant enzymes and treating with free-radical scavengers. Because mitochondria is the first resource of ROS in the cells, mitochondria-targeting therapies have also been developed to reduce OS.

Currently, tauopathies are still incurable. Since OS is a common hallmark for these diseases, antioxidants therapies have been prompted as potential treatments to delay, cure or prevent these diseases [130,131]. Antioxidant therapies are aimed to counteract the harmful effects of ROS and therefore prevent or treat OS-related diseases. OS is a lineal process that starts with an oxidant stressor and, if nothing prevents it, might end with cell damage (Figure 3). In the human body, antioxidants protect cells and organs by acting in three lines of defense during OS (Figure 3). Antioxidant enzymes such as SOD, CAT, GPX, and metal chelating proteins act in the first line of defense suppressing the generation of free radicals. Free radical-scavengers act as the second line of defense. These compounds interact with free radicals and neutralize them to prevent cell damage. They include vitamins, carotenoids, flavonoids, polyphenols and CoQ10 (Ubiquinol), among others. The third line of defense consists of restoring the impairments caused by free radicals. Several enzymes are involved in this line of defense and include lipases, proteases, DNA repair enzymes, and transferases. Antioxidant therapies research has been focused on targeting the first two lines of OS defense by activating antioxidant enzymes and treating with free-radical scavengers. Additionally, because mitochondria is the first resource of ROS in the cells, mitochondria-targeting therapies have also been developed to reduce OS (Figure 3). Below, we summarize the antioxidant strategies that have been proposed as a potential treatment for tauopathies.

### 4.1. Treatments Targeting Antioxidant Enzymes

The endogenous enzymes that prevent OS are essential for neuronal protection against oxidative damage and neuronal survival [27,132]. The levels and activity of some antioxidant enzymes are decreased in tauopathy patients [26,70] and, thus, the administration of treatments able to increase their levels and/or activity would be an ideal therapeutic approach for these diseases. Due to its instability and easy degradation in the gastrointestinal tract, the oral administration of enzymes for therapeutic applications is very challenging. Therefore, it has been proposed that the use of drugs able to indirectly activate the levels and activity of these enzymes might be a good therapeutic strategy. The expression of some antioxidant enzymes is regulated by transcription factors such as the nuclear factor-erythroid 2 (NF-E2) related factor 2 (Nrf2) [133,134]. The pharmacological targeting of Nrf2 with compounds such as benfotiamine, methylene blue or dimethyl fumarate has demonstrated good effects, reducing OS and tau pathogenesis in murine models of primary tauopathies [135,136,137] and other AD models [138]. The hormone melatonin has also been reported to stimulate the activity and expression of antioxidant enzymes such as nitric oxide synthase, glutathione peroxidase and superoxide dismutase [139,140,141]. Melatonin and its derivatives are also powerful direct free radical scavengers [142,143,144,145] and are implicated in the inhibition of pro-oxidant enzymes [146,147] and the enhancement of mitochondrial function [145,146]. In addition to the antioxidant properties, melatonin also has a direct role regulating GSK-3β activity and tau phosphorylation [148,149,150,151]. Due to these properties, melatonin has been proposed as a potentially useful agent in the prevention and treatment of tauopathies.

The above preclinical evidence suggests that the activation of antioxidant enzymes could be a potential antioxidant therapeutic strategy for tauopathies; however, further clinical trials are still needed to determine the clinical value and efficacy of these treatments.

### 4.2. Free Radical Scavengers

Free radical scavengers are antioxidants that act in the second line of antioxidant defense, preventing reactive oxygen species from being formed or removed them before they can damage vital components of the cell. Although the human body generates free radical scavengers, the majority of scavengers used in antioxidant therapies were synthesized by plants and, thus, diets supplemented with vegetables and herbs rich in these antioxidants could potentially prevent tau-related neurodegenerative diseases. The most exogenous free radical scavengers proposed to be used in antioxidant therapies are vitamins, carotenoids and polyphenols.

Vitamin E, C and carotenoids are potent exogenous antioxidants that protect against lipid peroxidation [152]. Several foods are rich in these antioxidants. Vitamin E is mostly found in plant-based oils, nuts, seeds, and vegetables (beet greens, collard greens, spinach, pumpkin, red bell pepper, asparagus, mango, avocado), whereas citrus fruits and cruciferous vegetables are the best sources of vitamin C. Carotenoid-enriched foods include yam, kale, spinach, watermelon, cantaloupe, bell pepper, tomato, carrot, mango and orange, among others. Although the three scavengers can be intake by diet, absorption in the intestine is different because vitamin E and carotenoids are lipid-soluble and vitamin C is soluble in water. All three antioxidants have demonstrated potentially good effects preventing tauopathies in preclinical studies; however, the majority of the studies have been performed using AD models and do not take account other tauopathies. Vitamin E (*α*-tocopherol) delayed the development of tau pathology and improved cognitive performance in rodent models of AD and other tauopathies [153,154]. Interestingly, vitamin E only reduced senile plaque deposition in mouse models when it was administered prior to the appearance of AD pathology [155]. Importantly, vitamin E treatment reduced neuronal damage and slowed the disease progression in AD patients [156]. Furthermore, it has been shown that AD patients whose diets included vitamin E tended to survive longer [157], suggesting that this vitamin may be beneficial to prevent AD. However, other clinical trials indicated that vitamin E did not have good effects decreasing OS in plasma [158,159] or improving the mini-mental state of AD patients [156]. It is important to highlight that the majority of preclinical studies and clinical trials explore the effectiveness of vitamin E in AD, but not in other tauopathies. Additional studies should be carried out to confirm if vitamin E is beneficial for the treatment of all tau-related diseases. Vitamin C (ascorbic acid) is a powerful antioxidant and free radical scavenger present in plasma and in the CNS [160,161]. Vitamin C has demonstrated a protective function against AD and other tauopathies [162,163]. The majority of studies about the effect of vitamin C in tauopathies have been related to AD and showed the positive effects of vitamin C reducing Aβ plaque deposition [164,165]. The effect of vitamin C reducing OS and neurodegeneration [126] and delaying tau pathogenesis [166] was also demonstrated in FTLD-Tau mouse models, suggesting that vitamin C could be a potential treatment for both primary tauopathies and AD. Although preclinical experiments showed vitamin C as a promising treatment for tauopathies, clinical trials have not shown good results [167,168]. The ability to transport ingested vitamin C from the intestines into blood is limited by the sodium-dependent vitamin C transporter (SVCT1) and, thus, high intakes might be not beneficial as it cannot be absorbed. Carotenoids such as β-carotene and lycopene are lipid-soluble antioxidants able to quench singlet oxygen rapidly [169]. The effect of carotenoids in tauopathies has been studied mostly in cellular and rodent models of AD. Both β-carotene and lycopene reduced the OS observed in these models [81,170,171,172,173], suggesting the potential use of these antioxidants as therapies for the treatment of AD and maybe other tauopathies. However, the positive effect of carotenoids in tauopathy patients has not yet been demonstrated. To summarize, although vitamin E, C and carotenoids antioxidant therapies have demonstrated success in preclinical studies, the effectiveness of these antioxidants in human preventive studies and clinical trials is still not clear [174]. Furthermore, it has been reported that a high dosage of vitamins and carotene supplements increased mortality and the risk of other diseases such as cancer or heart attack [175,176,177]. The lack of effectiveness in clinical trials, together with the potential toxicity of vitamins and carotenoids, suggest that these antioxidants should be used carefully.

Polyphenols are a diverse group of natural compounds whose chemical structure has one or more phenolic rings [178]. They are classified into two main categories, flavonoids and non-flavonoid compounds. In general, polyphenols are the main source of dietary antioxidants and usually are effortlessly absorbed in the intestine. Polyphenols are found in plant foods such as fruits, vegetables, and whole grains [179]. Some polyphenols such as resveratrol, curcumin, catechins and phenolic acids have demonstrated neuroprotective effects in tauopathies. The polyphenol that have demonstrated more impact in tauopathies, especially in AD, is resveratrol (3,5,4′-trihydroxystilbene) [12,180,181,182,183,184]. Resveratrol is a naturally occurring non-flavonoid polyphenol mostly found in grapes but also in peanuts and berries of the *vaccinium* species [185]. Both resveratrol isomers (cis- and trans) occur naturally, but the trans form seems to have higher neuroprotective activity [186]. In addition to being a potent antioxidant, resveratrol also has other mechanisms of action, including the inhibition of cyclooxygenase activity [187], ribonucleotide reductase [188], protein kinase C [189] and DNA polymerase [190], and also has antiestrogenic properties [191] and anti-platelet aggregation activity [192]. Furthermore, it activates sirtuin-1 (SIRT1), an NAD^+^-dependent protein deacetylase [193], and AMP kinase (AMPK) [194], an important glucose sensor that inhibits acetyl-CoA carboxylase, thereby increasing oxidation of fatty acids and decreasing their synthesis. Together, all these downstream targets may impact processes such as neuronal survival, mitochondrial biogenesis and prevention of protein aggregate formation, all of which contribute to the delay in symptoms and increased viability observed in tauopathy models after resveratrol treatment. Although resveratrol is a potent antioxidant, the most studied mechanism behind its neuroprotective role in tauopathies is the activation of the histone deacetylase SIRT1 [12,195,196]. Much evidence indicates that tau acetylation is an essential PTM that regulates tau pathogenesis. Tau acetylation at specific sites impaired tau degradation, leading to tau accumulation [12,196,197]. Furthermore, it was described that acetylation increased tau propensity to aggregate [75,198]. SIRT1 is one of the main deacetylases regulating tau acetylation. In tauopathy patients, tau protein is hyperacetylated and SIRT1 is downregulated [12,199]. Due to its role inhibiting tau acetylation, resveratrol has been proposed as a potential treatment for tauopathies and a good effect of resveratrol treatment in decreasing tau levels and rescuing the neuronal death characteristic of tauopathies has been reported in mouse and cellular models [12,200,201]. Several clinical trials have shown resveratrol to be safe and reasonably well-tolerated [202,203]. Furthermore, results from clinical trials in AD have provided evidence that resveratrol might be an effective treatment for AD [204,205]. However, further clinical trials in AD and other tauopathies should be performed to verify resveratrol effectiveness in treating these diseases. Since resveratrol has so many mechanistic targets, it is unknown if the effect of resveratrol treatment is related to its role as an antioxidant or as a tau acetylation inhibitor. Whatever the case may be, due to its multitarget characteristics, resveratrol is now considered one of the best candidates for the treatment of tauopathies.

Other non-flavonoid polyphenols such as curcumin (found in turmeric) and phenolic acids (including caffeine) have demonstrated good results in tauopathy models. For example, as it has been previously reviewed, curcumin (diferuloylmethane) is able to reduce OS, affect toxic protein aggregation, and protect against apoptosis in models of tauopathy [206,207]. Curcumin can modulate toxic tau aggregation in vitro [208] and in cell cultures [209]; however, the binding of curcumin to tau aggregates was not observed in post-mortem brain tissue sections from tauopathy patients [210]. While curcumin showed promising results in in vivo models of tauopathies [211,212,213], there is a challenge for in vivo use due to its poor absorption, fast metabolism, and rapid elimination. Several analogs have been designed to overcome the low oral bioavailability of curcumin [214]. Clinical studies about curcumin’s effects on cognitive deficits are mixed and important information regarding the effect of curcumin and analogs in bioavailability, safety, and tolerability, is lacking [215]. Homogenized clinical trials are needed to completely understand curcumin’s therapeutic potential. Phenolic acids are wildly distributed in natural sources (fruits, coffee, tea, and grains), have long stability in foods and show a high intestinal intake [216] and efficient brain absorption [217]. These compounds have shown good effects in models of AD [218]; however, the effect in primary tauopathies has not been studied. Due to their diverse neuroprotective effects, which have been extensively reviewed elsewhere [218,219], phenolic acids are considered potential candidates to treat tauopathies.

Flavonoids polyphenols such as catechins (found in Green tea), and in particular (−)epigallocatechin gallate (EGCG), have demonstrated antioxidant and anti-inflammatory effects on microglia and astrocytes [220]. EGCG decreased lipid peroxidation but had no effect on iron metabolism despite its presumed chelating abilities [221]. EGCG also has the potential to reduce protein aggregates [222] and it was demonstrated that catechins inhibited tau filament formation in a FTLD-tau model [223]. The effect of catechins in tau pathogenesis could be related to the effect of EGCG regulating phosphatidylinositol 3-kinase (PI3K/Akt) and GSK-3β kinases pathways [224]. Increased GSK-3β regulates tau phosphorylation and increased GSK-3β levels are associated with the formation of neurofibrillary tangles and neuronal death [225], thus catechins could regulate tau phosphorylation and pathogenesis by its role in regulating the GSK-3β pathway. Other flavonoids, such as luteolin (3′,4′,5,7-tetrahydroxyflavone) have also been tested in AD animal models [226,227] and have been demonstrated to improve the symptoms of patients with FTLD [228].

Because food is the main resource of free-radical scavenger antioxidants, diet could be essential in the prevention of tauopathies. Diet can also have other potential benefits for tauopathy patients; for example, the modification of gut microbiota. The microbiota is composed mainly of bacteria that colonize all mucosal surfaces of the gastrointestinal tract. It has been reported that microbiota changes could be a cause of different diseases including tauopathies such as AD [229,230,231,232]. Microbiota may also impact brain OS by elevating ROS levels or impairing the antioxidant system [233]. Furthermore, the microbiota also produces neurotoxic substances such as lipopolysaccharides and amyloid proteins, which can also reach to CNS and promote microglial activation and neuroinflammation, elevated ROS levels, and/or making neurons more susceptible to OS [234]. As a consequence of the link between microbiota, OS and neurodegeneration, the modification of the gut microbiota composition by diet may be helpful as a new preventive and therapeutic option for neurodegenerative diseases, specifically tauopathies. More research should be conducted to probe this hypothesis.

### 4.3. Treatments Targeting Mitochondria

Mitochondria is the main resource of ROS and mitochondrial dysfunction is a main characteristic of tauopathy patients [2,34,35,36,37]. Therefore, therapeutic strategies targeting mitochondria have been developed to treat tauopathies. The compounds targeting mitochondria are denominated metabolic antioxidants and include α-lipoic acid, coenzyme Q10 (CoQ10) and derivatives. Those antioxidants easily penetrate the cell to target the mitochondria and, thus, may provide the greatest protection. α-lipoic acid, also called thioctic acid, is a coenzyme of mitochondrial pyruvate dehydrogenase and *α*-ketoglutarate dehydrogenase. It also recycles other antioxidants such as vitamin C and E and glutathione, increases the production of acetylcholine or acts as a chelator of redox-active metals to combat the accumulation of lipid peroxidation products [235]. The effect of *α*-lipoic treatment has been studied in animal models of tauopathies. For example, α-lipoic acid was able to improve the abnormal behavior of P301S tau transgenic mice by mitigating OS and tauopathy [236]. Similarly, α-lipoic acid treatment ameliorated OS and rescued behavior deficits in a drosophila tauopathy model (tau^R406W^) [237]. Chronic administration of *α*-lipoic acid also protected against OS and reduced memory deficits in the Tg2576 AD mice model; however, it did not affect Aβ levels or plaque deposition [238]. CoQ10 (ubiquinone) is an important cofactor of the electron transport chain where it accepts electrons from complex I and II and, thus, preserves mitochondrial membrane potential during OS [239]. Both in vitro and in vivo studies have demonstrated the potential neuroprotective effect of CoQ10 in tauopathies. AD and FTLD preclinical studies suggest that CoQ10 could improve memory skills and cognitive abilities [240,241]. Furthermore, CoQ10 reduced tau phosphorylation in mouse hippocampal neurons after inducing cognitive deficiency with sevoflurane anesthesia [242]. CoQ10 is widely available in multiple formulations and is very well tolerated with minimal adverse effects [243], making it an attractive potential therapy. With this data in mind, several clinical studies of CoQ10 have been performed in tauopathy patients, with equivocal findings. Results from a clinical trial performed in patients suffering of the primary tauopathy PSP showed that CoQ10 treatment slightly improved the PSP rating scale [244]. On the other hand, clinical studies in AD did not support CoQ10′s potential to promote cognitive function or prevent dementia [168]. Interestingly, it was reported that CoQ10 induced tau aggregation, tau filaments, and Hirano bodies [245]. These contradictory data confirm that more research should be carried out to further ascertain the effects of CoQ10 in different tauopathies. Mito Q, a CoQ10 derivative produced by the conjugation of the lipophilic triphenylphosphonium (TPP^+^) cation to CoQ10 [246], was also explored as a treatment for tauopathies. MitoQ seems to be a more effective antioxidant than the regular CoQ10 because it is better absorbed by the mitochondria. As its precursor, MitoQ exerted protective effects on cells by reducing free radicals, decreasing oxidative damage, and maintaining mitochondrial functions. MitoQ prevented neurotoxicity in models of AD [247] and its benefits were also demonstrated in iPSC-derived neurons from FTLD-TAU patients carrying the 10 + 16 *MAPT* mutation [36,248]. These reports suggest that MitoQ may be a candidate drug to treat tauopathy patients. Several ongoing clinical trials are testing the efficacy of MitoQ for the treatment of neurodegenerative diseases and, thus, we need to wait to know the potential benefits of MitoQ.

Based on the outcomes from the experiments exposed in this section, it has been suggested that antioxidants could be used to treat tauopathies. However, it is important to highlight that the use of antioxidants also have some limitations such as the low bioavailability of some antioxidants (e.g., polyphenols, curcumin, resveratrol, catechins) [249,250] and the toxicity of some vitamins (A, D or E) when used at high concentrations [251].

## 5. Present and Future of Antioxidant Therapies

Although preclinical studies pointed to antioxidant substances as potential therapeutic agents for the treatment of tauopathies, the translation of these preclinical studies into clinical therapeutic strategies has not yet led to significant advances [252]. The failure of the current antioxidant clinical trials for tauopathies could be associated with several reasons.

For example, the inappropriate design of some clinical trials might be affecting their outcomes. It is plausible that some clinical trials have used an insufficient dose of the chosen antioxidant or have not taken into account the limited solubility of some antioxidants. Furthermore, unsuitable timing (e.g., too late in the disease) or inappropriate duration for the treatment might also affect the efficiency of the treatments. Improvements in the design of clinical trials will facilitate the validation of the use of antioxidants to treat tauopathies.

Another reason for the failure of antioxidant clinical trials might be the insufficient knowledge of antioxidants’ blood–brain barrier (BBB) penetration. Although some antioxidants such as vitamin C, α-lipoic acid or resveratrol have been described to cross the BBB, others demonstrate poor BBB penetrability, or it is still unknown [253,254]. To facilitate the delivery of antioxidant therapies across the BBB, it is necessary to design BBB-penetrable antioxidants by inducing chemical modifications, as well as using nanoplatforms and/or new administration routes.

The low efficiency of antioxidant clinical trials could also be associated with the fact that tauopathies, as other neurological syndromes, are multifactorial diseases in which several molecular mechanisms contribute to the disease and, perhaps, oxidative damage may not be the main cause contributing to the pathophysiology of tauopathies. To solve this problem, future therapeutic approaches should consider using combinations of different antioxidants or combine antioxidants with compounds targeting other molecular mechanisms.

The bad translation of preclinical studies to clinical trials could also be associated with the limitations of the models used in preclinical studies. In this regard, current cellular and animal tauopathy models do not completely recapitulate the complexity of the human brain [255,256] and, thus, the results obtained using these models cannot be completely translated to patients. More accurate tauopathy preclinical models such as the novel brain organoids [257] should be used to confirm previous preclinical antioxidant research.

The failure of antioxidant clinical trials could also be associated with the fact that the altered molecular mechanisms leading to OS might vary between different tauopathies. In this regard, although preclinical studies have been performed using several tauopathy models, the majority of completed antioxidant clinical trials have been carried out on AD patients. An updated list of concluded antioxidant clinical trials in tauopathies can be found on the US National Library of Medicine website (clinicaltrials.gov). New clinical trials recruiting primary tauopathy patients should be conducted to validate the effectiveness of antioxidant treatment tauopathies.

Despite the poor results of clinical trials, preclinical studies have proved that antioxidants have the potential to be beneficial for disease prevention and healthy aging. The majority of antioxidants are natural compounds present in food. Following the notion that “prevention is better than cure”, a balanced antioxidant-enriched diet will help to prevent or delay the onset of tauopathies.

## 6. Concluding Remarks

Although more research should be conducted to completely understand the etiology of tauopathies, OS has emerged as one of the multiple molecular mechanisms involved in the pathophysiology of tau-related diseases. Currently, it is still unknown if tau accumulation is a cause or a consequence of OS and it has been speculated that tau pathology and OS could be two elements of a “vicious circle”, where OS might trigger tau pathology, which, as a consequence, will later promote more OS (Figure 2).

Current research efforts are focused on the identification and development of effective therapies to treat tauopathies. Based on preclinical studies, antioxidant therapies have emerged as good candidates to treat tauopathies; however, antioxidant clinical trials have not shown good results. Unraveling the molecular mechanisms underlying OS in tauopathies would contribute to the development of more efficient therapies to treat and/or prevent these devastating neurodegenerative diseases.

## References

[1] Das K., Roychoudhury A. (2014). Reactive oxygen species (ROS) and response of antioxidants as ROS-scavengers during environmental stress in plants. Front. Environ. Sci..

[2] Brand M.D., Affourtit C., Esteves T.C., Green K., Lambert A.J., Miwa S., Pakay J.L., Parker N. (2004). Mitochondrial superoxide: Production, biological effects, and activation of uncoupling proteins. Free Radic. Biol. Med..

[3] Finkel T. (2011). Signal transduction by reactive oxygen species. J. Cell Biol..

[4] Dröge W. (2002). Free Radicals in the Physiological Control of Cell Function. Physiol. Rev..

[5] Auten R.L., Davis J.M. (2009). Oxygen Toxicity and Reactive Oxygen Species: The Devil Is in the Details. Pediatr. Res..

[6] Yang H.-Y., Lee T.-H. (2015). Antioxidant enzymes as redox-based biomarkers: A brief review. BMB Rep..

[7] Mirończuk-Chodakowska I., Witkowska A.M., Zujko M.E. (2018). Endogenous non-enzymatic antioxidants in the human body. Adv. Med. Sci..

[8] Forman H.J., Zhang H. (2021). Targeting oxidative stress in disease: Promise and limitations of antioxidant therapy. Nat. Rev. Drug Discov..

[9] Arendt T., Stieler J.T., Holzer M. (2016). Tau and tauopathies. Brain Res. Bull..

[10] De Ture M.A., Dickson D.W. (2019). The neuropathological diagnosis of Alzheimer’s disease. Mol. Neurodegener..

[11] Chung D.-E.C., Roemer S., Petrucelli L., Dickson D.W. (2021). Cellular and pathological heterogeneity of primary tauopathies. Mol. Neurodegener..

[12] Alquezar C., Schoch K.M., Geier E.G., Ramos E.M., Scrivo A., Li K.H., Argouarch A.R., Mlynarski E.E., Dombroski B., De Ture M. (2021). TSC1 loss increases risk for tauopathy by inducing tau acetylation and preventing tau clearance via chaperone-mediated autophagy. Sci. Adv..

[13] Kovacs G.G. (2018). Tauopathies. Handbook of Clinical Neurology.

[14] Salim S. (2017). Oxidative Stress and the Central Nervous System. J. Pharmacol. Exp. Ther..

[15] Bullmore E., Sporns O. (2012). The economy of brain network organization. Nat. Rev. Neurosci..

[16] Baxter P.S., Hardingham G.E. (2016). Adaptive regulation of the brain’s antioxidant defences by neurons and astrocytes. Free Radic. Biol. Med..

[17] Di Pietro V., Lazzarino G., Amorini A.M., Tavazzi B., D’Urso S., Longo S., Vagnozzi R., Signoretti S., Clementi E., Giardina B. (2014). Neuroglobin expression and oxidant/antioxidant balance after graded traumatic brain injury in the rat. Free Radic. Biol. Med..

[18] Feuerstein D., Backes H., Gramer M., Takagaki M., Gabel P., Kumagai T., Graf R. (2016). Regulation of cerebral metabolism during cortical spreading depression. J. Cereb. Blood Flow Metab..

[19] Castellani R., Smith M., Richey P., Kalaria R., Gambetti P., Perry G. (1995). Evidence for oxidative stress in Pick disease and corticobasal degeneration. Brain Res..

[20] Dumont M., Stack C., Elipenahli C., Jainuddin S., Gerges M., Starkova N.N., Yang L., Starkov A.A., Beal F. (2011). Behavioral deficit, oxidative stress, and mitochondrial dysfunction precede tau pathology in P301S transgenic mice. FASEB J..

[21] Martínez A., Carmona M., Portero-Otin M., Naudí A., Pamplona R., Ferrer I. (2008). Type-Dependent Oxidative Damage in Frontotemporal Lobar Degeneration: Cortical Astrocytes Are Targets of Oxidative Damage. J. Neuropathol. Exp. Neurol..

[22] Litvan I. (2004). Update on progressive supranuclear palsy. Curr. Neurol. Neurosci. Rep..

[23] Odetti P., Garibaldi S., Norese R., Angelini G., Marinelli L., Valentini S., Menini S., Traverso N., Zaccheo D., Siedlak S. (2000). Lipoperoxidation Is Selectively Involved in Progressive Supranuclear Palsy. J. Neuropathol. Exp. Neurol..

[24] Albers D.S., Augood S.J., Martin D.M., Standaert D.G., Vonsattel J.P.G., Beal M.F. (1999). Evidence for Oxidative Stress in the Subthalamic Nucleus in Progressive Supranuclear Palsy. J. Neurochem..

[25] Cantuti-Castelvetri I., Keller-McGandy C.E., Albers D.S., Beal M.F., Vonsattel J.-P., Standaert D.G., Augood S.J. (2002). Expression and activity of antioxidants in the brain in progressive supranuclear palsy. Brain Res..

[26] Aoyama K., Matsubara K., Kobayashi S. (2006). Aging and oxidative stress in progressive supranuclear palsy. Eur. J. Neurol..

[27] Nunomura A., Castellani R.J., Zhu X., Moreira P.I., Perry G., Smith M.A. (2006). Involvement of Oxidative Stress in Alzheimer Disease. J. Neuropathol. Exp. Neurol..

[28] Wojsiat J., Zoltowska K.M., Laskowska-Kaszub K., Wojda U. (2018). Oxidant/Antioxidant Imbalance in Alzheimer’s Disease: Therapeutic and Diagnostic Prospects. Oxidative Med. Cell. Longev..

[29] Behl C. (1999). Alzheimer’s disease and oxidative stress: Implications for novel therapeutic approaches. Prog. Neurobiol..

[30] Chauhan V., Chauhan A. (2006). Oxidative stress in Alzheimer’s disease. Pathophysiology.

[31] Pohanka M. (2014). Alzheimer´s Disease and Oxidative Stress: A Review. Curr. Med. Chem..

[32] Llanos-González E., Henares-Chavarino Á.A., Pedrero-Prieto C.M., García-Carpintero S., Frontiñán-Rubio J., Sancho-Bielsa F.J., Alcain F.J., Peinado J.R., Rabanal-Ruíz Y., Durán-Prado M. (2020). Interplay Between Mitochondrial Oxidative Disorders and Proteostasis in Alzheimer’s Disease. Front. Neurosci..

[33] Tamagno E., Guglielmotto M., Vasciaveo V., Tabaton M. (2021). Oxidative Stress and Beta Amyloid in Alzheimer’s Disease. Which Comes First: The Chicken or the Egg?. Antioxidants.

[34] Castellani R., Hirai K., Aliev G., Drew K.L., Nunomura A., Takeda A., Cash A.D., Obrenovich M.E., Perry G., Smith M.A. (2002). Role of mitochondrial dysfunction in Alzheimer’s disease. J. Neurosci. Res..

[35] Wang W., Zhao F., Ma X., Perry G., Zhu X. (2020). Mitochondria dysfunction in the pathogenesis of Alzheimer’s disease: Recent advances. Mol. Neurodegener..

[36] Esteras N., Rohrer J., Hardy J., Wray S., Abramov A.Y. (2017). Mitochondrial hyperpolarization in iPSC-derived neurons from patients of FTDP-17 with 10+16 MAPT mutation leads to oxidative stress and neurodegeneration. Redox Biol..

[37] Albers D.S., Beal M.F. (2002). Mitochondrial dysfunction in progressive supranuclear palsy. Neurochem. Int..

[38] Beal M.F. (1996). Mitochondria, free radicals, and neurodegeneration. Curr. Opin. Neurobiol..

[39] Hirai K., Aliev G., Nunomura A., Fujioka H., Russell R.L., Atwood C.S., Johnson A.B., Kress Y., Vinters H.V., Tabaton M. (2001). Mitochondrial Abnormalities in Alzheimer’s Disease. J. Neurosci..

[40] Zhu X., Smith M.A., Perry G., Aliev G. (2004). Mitochondrial failures in Alzheimer’s disease. Am. J. Alzheimers Dis. Other Dement..

[41] Rhein V., Song X., Wiesner A., Ittner L.M., Baysang G., Meier F., Ozmen L., Bluethmann H., Dröse S., Brandt U. (2009). Amyloid-β and tau synergistically impair the oxidative phosphorylation system in triple transgenic Alzheimer’s disease mice. Proc. Natl. Acad. Sci. USA.

[42] Yao J., Irwin R.W., Zhao L., Nilsen J., Hamilton R.T., Brinton R.D. (2009). Mitochondrial bioenergetic deficit precedes Alzheimer’s pathology in female mouse model of Alzheimer’s disease. Proc. Natl. Acad. Sci. USA.

[43] Ittner L.M., Fath T., Ke Y.D., Bi M., van Eersel J., Li K.M., Gunning P., Götz J. (2008). Parkinsonism and impaired axonal transport in a mouse model of frontotemporal dementia. Proc. Natl. Acad. Sci. USA.

[44] Kopeikina K.J., Carlson G.A., Pitstick R., Ludvigson A.E., Peters A., Luebke J.I., Koffie R.M., Frosch M.P., Hyman B.T., Spires-Jones T.L. (2011). Tau Accumulation Causes Mitochondrial Distribution Deficits in Neurons in a Mouse Model of Tauopathy and in Human Alzheimer’s Disease Brain. Am. J. Pathol..

[45] Rodríguez-Martín T., Pooler A.M., Lau D.H., Mórotz G.M., De Vos K.J., Gilley J., Coleman M.P., Hanger D.P. (2016). Reduced number of axonal mitochondria and tau hypophosphorylation in mouse P301L tau knockin neurons. Neurobiol. Dis..

[46] Cummins N., Tweedie A., Zuryn S., Bertran-Gonzalez J., Götz J. (2019). Disease-associated tau impairs mitophagy by inhibiting Parkin translocation to mitochondria. EMBO J..

[47] David D., Hauptmann S., Scherping I., Schuessel K., Keil U., Rizzu P., Ravid R., Dröse S., Brandt U., Müller W.E. (2005). Proteomic and Functional Analyses Reveal a Mitochondrial Dysfunction in P301L Tau Transgenic Mice. J. Biol. Chem..

[48] Hu Y., Li X.-C., Wang Z.-H., Luo Y., Zhang X., Liu X.-P., Feng Q., Wang Q., Yue Z., Chen Z. (2016). Tau accumulation impairs mitophagy *via* increasing mitochondrial membrane potential and reducing mitochondrial Parkin. Oncotarget.

[49] Li X.-C., Hu Y., Wang Z.-H., Luo Y., Zhang Y., Liu X.-P., Feng Q., Wang Q., Ye K., Liu G.-P. (2016). Human wild-type full-length tau accumulation disrupts mitochondrial dynamics and the functions via increasing mitofusins. Sci. Rep..

[50] Tracy T.E., Madero-Pérez J., Swaney D.L., Chang T.S., Moritz M., Konrad C., Ward M.E., Stevenson E., Hüttenhain R., Kauwe G. (2022). Tau interactome maps synaptic and mitochondrial processes associated with neurodegeneration. Cell.

[51] DuBoff B., Götz J., Feany M.B. (2012). Tau Promotes Neurodegeneration via DRP1 Mislocalization In Vivo. Neuron.

[52] Dubey M., Chaudhury P., Kabiru H., Shea T.B. (2008). Tau inhibits anterograde axonal transport and perturbs stability in growing axonal neurites in part by displacing kinesin cargo: Neurofilaments attenuate tau-mediated neurite instability. Cell Motil. Cytoskelet..

[53] Eckert A., Nisbet R., Grimm A., Götz J. (2014). March separate, strike together—Role of phosphorylated TAU in mitochondrial dysfunction in Alzheimer’s disease. Biochim. Biophys. Acta.

[54] Stamer K., Vogel R., Thies E., Mandelkow E., Mandelkow E.-M. (2002). Tau blocks traffic of organelles, neurofilaments, and APP vesicles in neurons and enhances oxidative stress. J. Cell Biol..

[55] Petersen J.D., Kaech S., Banker G. (2014). Selective Microtubule-Based Transport of Dendritic Membrane Proteins Arises in Concert with Axon Specification. J. Neurosci..

[56] Gendron T.F., Petrucelli L. (2009). The role of tau in neurodegeneration. Mol. Neurodegener..

[57] Kandimalla R., Manczak M., Yin X., Wang R., Reddy P.H. (2017). Hippocampal phosphorylated tau induced cognitive decline, dendritic spine loss and mitochondrial abnormalities in a mouse model of Alzheimer’s disease. Hum. Mol. Genet..

[58] Schulz K.L., Eckert A., Rhein V., Mai S., Haase W., Reichert A.S., Jendrach M., Müller W.E., Leuner K. (2012). A New Link to Mitochondrial Impairment in Tauopathies. Mol. Neurobiol..

[59] Grimm A., Biliouris E.E., Lang U.E., Götz J., Mensah-Nyagan A.G., Eckert A. (2016). Sex hormone-related neurosteroids differentially rescue bioenergetic deficits induced by amyloid-β or hyperphosphorylated tau protein. Cell Mol. Life Sci..

[60] Li H.-L., Wang H.-H., Liu S.-J., Deng Y.-Q., Zhang Y.-J., Tian Q., Wang X.-C., Chen X.-Q., Yang Y., Zhang J.-Y. (2007). Phosphorylation of tau antagonizes apoptosis by stabilizing β-catenin, a mechanism involved in Alzheimer’s neurodegeneration. Proc. Natl. Acad. Sci. USA.

[61] Jara C., Aránguiz A., Cerpa W., Tapia-Rojas C., Quintanilla R.A. (2018). Genetic ablation of tau improves mitochondrial function and cognitive abilities in the hippocampus. Redox Biol..

[62] Quinn J.P., Corbett N.J., Kellett K.A.B., Hooper N.M. (2018). Tau Proteolysis in the Pathogenesis of Tauopathies: Neurotoxic Fragments and Novel Biomarkers. J. Alzheimers Dis..

[63] Atlante A., Amadoro G., Bobba A., de Bari L., Corsetti V., Pappalardo G., Marra E., Calissano P., Passarella S. (2008). A peptide containing residues 26–44 of tau protein impairs mitochondrial oxidative phosphorylation acting at the level of the adenine nucleotide translocator. Biochim. Biophys. Acta.

[64] Amadoro G., Corsetti V., Atlante A., Florenzano F., Capsoni S., Bussani R., Mercanti D., Calissano P. (2011). Interaction between NH(2)-Tau Fragment and Aβ in Alzheimer’s disease mitochondria contributes to the synaptic deterioration. Neurobiol. Aging.

[65] Quintanilla R.A., von Bernhardi R., Godoy J.A., Inestrosa N.C., Johnson G.V.W. (2014). Phosphorylated tau potentiates Aβ-induced mitochondrial damage in mature neurons. Neurobiol. Dis..

[66] Nunomura A., Perry G., Aliev G., Hirai K., Takeda A., Balraj E.K., Jones P.K., Ghanbari H., Wataya T., Shimohama S. (2001). Oxidative Damage Is the Earliest Event in Alzheimer Disease. J. Neuropathol. Exp. Neurol..

[67] Kozakiewicz M., Kornatowski M., Krzywińska O., Kędziora-Kornatowska K. (2019). Changes in the blood antioxidant defense of advanced age people. Clin. Interv. Aging.

[68] Rybka J., Kupczyk D., Kędziora-Kornatowska K., Pawluk H., Czuczejko J., Szewczyk-Golec K., Kozakiewicz M., Antonioli M., Carvalho L.A., Kędziora J. (2011). Age-related changes in an antioxidant defense system in elderly patients with essential hypertension compared with healthy controls. Redox Rep..

[69] Andriollo-Sanchez M., Hininger-Favier I., Meunier N., Venneria E., O’Connor J.M., Maiani G., Coudray C., Roussel A.M. (2005). Age-related oxidative stress and antioxidant parameters in middle-aged and older European subjects: The ZENITH study. Eur. J. Clin. Nutr..

[70] Marcus D.L., Thomas C., Rodriguez C., Simberkoff K., Tsai J.S., Strafaci J.A., Freedman M.L. (1998). Increased Peroxidation and Reduced Antioxidant Enzyme Activity in Alzheimer’s Disease. Exp. Neurol..

[71] Fracassi A., Marcatti M., Zolochevska O., Tabor N., Woltjer R., Moreno S., Taglialatela G. (2021). Oxidative Damage and Antioxidant Response in Frontal Cortex of Demented and Nondemented Individuals with Alzheimer’s Neuropathology. J. Neurosci..

[72] Melov S., Adlard P.A., Morten K., Johnson F., Golden T.R., Hinerfeld D., Schilling B., Mavros C., Masters C.L., Volitakis I. (2007). Mitochondrial Oxidative Stress Causes Hyperphosphorylation of Tau. PLoS ONE.

[73] Dias-Santagata D., Fulga T.A., Duttaroy A., Feany M.B. (2007). Oxidative stress mediates tau-induced neurodegeneration in Drosophila. J. Clin. Investig..

[74] Fitzpatrick A.W.P., Falcon B., He S., Murzin A.G., Murshudov G., Garringer H.J., Crowther R.A., Ghetti B., Goedert M., Scheres S.H.W. (2017). Cryo-EM structures of tau filaments from Alzheimer’s disease. Nature.

[75] Arakhamia T., Lee C.E., Carlomagno Y., Duong D.M., Kundinger S.R., Wang K., Williams D., De Ture M., Dickson D.W., Cook C.N. (2020). Posttranslational Modifications Mediate the Structural Diversity of Tauopathy Strains. Cell.

[76] Falcon B., Noad J., McMahon H., Randow F., Goedert M. (2018). Galectin-8–mediated selective autophagy protects against seeded tau aggregation. J. Biol. Chem..

[77] Falcon B., Zivanov J., Zhang W., Murzin A.G., Garringer H.J., Vidal R., Crowther R.A., Newell K.L., Ghetti B., Goedert M. (2019). Novel tau filament fold in chronic traumatic encephalopathy encloses hydrophobic molecules. Nature.

[78] Mondragón-Rodríguez S., Perry G., Zhu X., Moreira P., Acevedo-Aquino M.C., Williams S. (2013). Phosphorylation of Tau Protein as the Link between Oxidative Stress, Mitochondrial Dysfunction, and Connectivity Failure: Implications for Alzheimer’s Disease. Oxidative Med. Cell. Longev..

[79] Alavi Naini S.M., Soussi-Yanicostas N. (2015). Tau hyperphosphorylation and oxidative stress, a critical vicious circle in neurodegenerative tauopathies?. Oxidative Med. Cell. Longev..

[80] Liu Z., Li T., Li P., Wei N., Zhao Z., Liang H., Ji X., Chen W., Xue M., Wei J. (2015). The Ambiguous Relationship of Oxidative Stress, Tau Hyperphosphorylation, and Autophagy Dysfunction in Alzheimer’s Disease. Oxidative Med. Cell. Longev..

[81] Liu T., Liu W.-H., Zhao J.-S., Meng F.-Z., Wang H. (2017). Lutein protects against β-amyloid peptide-induced oxidative stress in cerebrovascular endothelial cells through modulation of Nrf-2 and NF-κb. Cell Biol. Toxicol..

[82] Alquezar C., Arya S., Kao A.W. (2020). Tau Post-translational Modifications: Dynamic Transformers of Tau Function, Degradation, and Aggregation. Front. Neurol..

[83] Finelli M.J. (2020). Redox Post-translational Modifications of Protein Thiols in Brain Aging and Neurodegenerative Conditions—Focus on S-Nitrosation. Front. Aging Neurosci..

[84] Xiang W., Weisbach V., Sticht H., Seebahn A., Bussmann J., Zimmermann R., Becker C.-M. (2013). Oxidative stress-induced posttranslational modifications of human hemoglobin in erythrocytes. Arch. Biochem. Biophys..

[85] Alonso A.D.C., Zaidi T., Novak M., Grundke-Iqbal I., Iqbal K. (2001). Hyperphosphorylation induces self-assembly of τ into tangles of paired helical filaments/straight filaments. Proc. Natl. Acad. Sci. USA.

[86] Biernat J., Mandelkow E.-M. (1999). The Development of Cell Processes Induced by tau Protein Requires Phosphorylation of Serine 262 and 356 in the Repeat Domain and Is Inhibited by Phosphorylation in the Proline-rich Domains. Mol. Biol. Cell.

[87] Drewes G., Trinczek B., Illenberger S., Biernat J., Schmitt-Ulms G., Meyer H.E., Mandelkow E.M., Mandelkow E. (1995). Microtubule-Associated Protein/Microtubule Affinity-Regulating Kinase (P110(Mark)). A Novel Protein Kinase That Regulates Tau-Microtubule Interactions and Dynamic Instability by Phosphorylation at the Alzheimer- Specific Site Serine 262. J. Biol. Chem..

[88] Biernat J., Gustke N., Drewes G., Mandelkow E. (1993). Phosphorylation of Ser262 strongly reduces binding of tau to microtubules: Distinction between PHF-like immunoreactivity and microtubule binding. Neuron.

[89] Patil S., Chan C. (2005). Palmitic and stearic fatty acids induce Alzheimer-like hyperphosphorylation of tau in primary rat cortical neurons. Neurosci. Lett..

[90] Su B., Wang X., Lee H.G., Tabaton M., Perry G., Smith M.A., Zhu X. (2010). Chronic oxidative stress causes increased tau phosphorylation in M17 neuroblastoma cells. Neurosci. Lett..

[91] Ibáñez-Salazar A., Bañuelos-Hernandez B., Rodriguez-Leyva I., Chi-Ahumada E., Monreal-Escalante E., Jiménez-Capdeville M.E., Rosales-Mendoza S. (2017). Oxidative Stress Modifies the Levels and Phosphorylation State of Tau Protein in Human Fibroblasts. Front. Neurosci..

[92] Goedert M., Hasegawa M., Jakes R., Lawler S., Cuenda A., Cohen P. (1997). Phosphorylation of microtubule-associated protein tau by stress-activated protein kinases. FEBS Lett..

[93] Atzori C., Ghetti B., Piva R., Srinivasan A.N., Zolo P., Delisle M.B., Mirra S.S., Migheli A. (2001). Activation of the JNK/p38 Pathway Occurs in Diseases Characterized by Tau Protein Pathology and Is Related to Tau Phosphorylation But Not to Apoptosis. J. Neuropathol. Exp. Neurol..

[94] Tamagno E., Parola M., Bardini P., Piccini A., Borghi R., Guglielmotto M., Santoro G., Davit A., Danni O., Smith M.A. (2005). β-Site APP cleaving enzyme up-regulation induced by 4-hydroxynonenal is mediated by stress-activated protein kinases pathways. J. Neurochem..

[95] Elgenaidi I.S., Spiers J.P. (2019). Regulation of the phosphoprotein phosphatase 2A system and its modulation during oxidative stress: A potential therapeutic target?. Pharmacol. Ther..

[96] Lovell M.A., Xiong S., Xie C., Davies P., Markesbery W.R. (2004). Induction of hyperphosphorylated tau in primary rat cortical neuron cultures mediated by oxidative stress and glycogen synthase kinase-3. J. Alzheimers Dis..

[97] Feng Y., Xia Y., Yu G., Shu X., Ge H., Zeng K., Wang J., Wang X. (2013). Cleavage of GSK-3β by calpain counteracts the inhibitory effect of Ser9 phosphorylation on GSK-3β activity induced by H_2_O_2_. J. Neurochem..

[98] Lloret A., Badia M.-C., Giraldo E., Ermak G., Alonso M.-D., Pallardó F.V., Davies K.J.A., Viña J. (2011). Amyloid-β Toxicity and Tau Hyperphosphorylation are Linked Via RCAN1 in Alzheimer’s Disease. J. Alzheimers Dis..

[99] Lee K.-Y., Koh S.-H., Noh M.Y., Park K.-W., Lee Y.J., Kim S.H. (2007). Glycogen synthase kinase-3β activity plays very important roles in determining the fate of oxidative stress-inflicted neuronal cells. Brain Res..

[100] Kang S.-W., Kim S.J., Kim M.-S. (2017). Oxidative stress with tau hyperphosphorylation in memory impaired 1,2-diacetylbenzene-treated mice. Toxicol. Lett..

[101] Giraldo E., Lloret A., Fuchsberger T., Vina J. (2014). Aβ and tau toxicities in Alzheimer’s are linked via oxidative stress-induced p38 activation: Protective role of vitamin E. Redox Biol..

[102] Chen L., Liu L., Huang S. (2008). Cadmium activates the mitogen-activated protein kinase (MAPK) pathway via induction of reactive oxygen species and inhibition of protein phosphatases 2A and 5. Free Radic. Biol. Med..

[103] Cho M.-H., Kim D.-H., Choi J.-E., Chang E.-J., Yoon S.Y. (2012). Increased phosphorylation of dynamin-related protein 1 and mitochondrial fission in okadaic acid-treated neurons. Brain Res..

[104] Poppek D., Keck S., Ermak G., Jung T., Stolzing A., Ullrich O., Davies K.J.A., Grune T. (2006). Phosphorylation inhibits turnover of the tau protein by the proteasome: Influence of *RCAN1* and oxidative stress. Biochem. J..

[105] Zambrano C.A., Egaña J.T., Núñez M.T., Maccioni R.B., González-Billault C. (2004). Oxidative Stress Promotes Tau Dephosphorylation in Neuronal Cells: The Roles of Cdk5 and PP1. Free Radic. Biol. Med..

[106] Gamblin T.C., King M.E., Kuret J., Berry R.W., Binder L.I. (2000). Oxidative Regulation of Fatty Acid-Induced Tau Polymerization. Biochemistry.

[107] Pérez M., Cuadros R., Smith M.A., Perry G., Avila J. (2000). Phosphorylated, but not native, tau protein assembles following reaction with the lipid peroxidation product, 4-hydroxy-2-nonenal. FEBS Lett..

[108] Liu Q., Smith M.A., Avilá J., DeBernardis J., Kansal M., Takeda A., Zhu X., Nunomura A., Honda K., Moreira P. (2005). Alzheimer-specific epitopes of tau represent lipid peroxidation-induced conformations. Free Radic. Biol. Med..

[109] Gómez-Ramos A., Diaz-Nido J., Smith M.A., Perry G., Avila J. (2003). Effect of the lipid peroxidation product acrolein on tau phosphorylation in neural cells. J. Neurosci. Res..

[110] Sultana R., Boyd-Kimball D., Poon H.F., Cai J., Pierce W.M., Klein J.B., Markesbery W.R., Zhou X.Z., Lu K.P., Butterfield D.A. (2006). Oxidative modification and down-regulation of Pin1 in Alzheimer’s disease hippocampus: A redox proteomics analysis. Neurobiol. Aging.

[111] Hamdane M., Dourlen P., Bretteville A., Sambo A.-V., Ferreira S., Ando K., Kerdraon O., Bégard S., Geay L., Lippens G. (2006). Pin1 allows for differential Tau dephosphorylation in neuronal cells. Mol. Cell. Neurosci..

[112] Kimura T., Tsutsumi K., Taoka M., Saito T., Masuda-Suzukake M., Ishiguro K., Plattner F., Uchida T., Isobe T., Hasegawa M. (2013). Isomerase Pin1 Stimulates Dephosphorylation of Tau Protein at Cyclin-dependent Kinase (Cdk5)-dependent Alzheimer Phosphorylation Sites. J. Biol. Chem..

[113] Chen C.-H., Li W., Sultana R., You M.-H., Kondo A., Shahpasand K., Kim B.M., Luo M.-L., Nechama M., Lin Y.-M. (2015). Pin1 cysteine-113 oxidation inhibits its catalytic activity and cellular function in Alzheimer’s disease. Neurobiol. Dis..

[114] Newsholme P., Keane K.N., Carlessi R., Cruzat V. (2019). Oxidative stress pathways in pancreatic β-cells and insulin-sensitive cells and tissues: Importance to cell metabolism, function, and dysfunction. Am. J. Physiol. Cell Physiol..

[115] Gonçalves R.A., Wijesekara N., Fraser P.E., De Felice F.G. (2019). The Link Between Tau and Insulin Signaling: Implications for Alzheimer’s Disease and Other Tauopathies. Front. Cell. Neurosci..

[116] Zhang W., Xiao S., Ahn D.U. (2013). Protein Oxidation: Basic Principles and Implications for Meat Quality. Crit. Rev. Food Sci. Nutr..

[117] Schweers O., Mandelkow E.M., Biernat J., Mandelkow E. (1995). Oxidation of cysteine-322 in the repeat domain of microtubule-associated protein tau controls the in vitro assembly of paired helical filaments. Proc. Natl. Acad. Sci. USA.

[118] Caballero B., Wang Y., Diaz A., Tasset I., Juste Y.R., Stiller B., Mandelkow E.-M., Mandelkow E., Cuervo A.M. (2018). Interplay of pathogenic forms of human tau with different autophagic pathways. Aging Cell.

[119] Esteras N., Kundel F., Amodeo G.F., Pavlov E.V., Klenerman D., Abramov A.Y. (2021). Insoluble tau aggregates induce neuronal death through modification of membrane ion conductance, activation of voltage-gated calcium channels and NADPH oxidase. FEBS J..

[120] Simpson D.S.A., Oliver P.L. (2020). ROS Generation in Microglia: Understanding Oxidative Stress and Inflammation in Neurodegenerative Disease. Antioxidants.

[121] Asai H., Ikezu S., Tsunoda S., Medalla M., Luebke J., Haydar T., Wolozin B., Butovsky O., Kügler S., Ikezu T. (2015). Depletion of microglia and inhibition of exosome synthesis halt tau propagation. Nat. Neurosci..

[122] Bolós M., Llorens-Martín M., Jurado-Arjona J., Hernández F., Rábano A., Avila J. (2016). Direct Evidence of Internalization of Tau by Microglia In Vitro and In Vivo. J. Alzheimers Dis..

[123] Fasulo L., Visintin M., Novak M., Cattaneo A. (1998). Tau Truncation in Alzheimer’s Disease: Expression of a Fragment Encompassing PHF Core. Alzheimers Rep..

[124] Guillozet-Bongaarts A.L., Garcia-Sierra F., Reynolds M.R., Horowitz P.M., Fu Y., Wang T., Cahill M.E., Bigio E.H., Berry R.W., Binder L.I. (2005). Tau truncation during neurofibrillary tangle evolution in Alzheimer’s disease. Neurobiol. Aging.

[125] Paholikova K., Salingova B., Opattova A., Skrabana R., Majerova P., Zilka N., Kovacech B., Zilkova M., Barath P., Novak M. (2015). N-terminal Truncation of Microtubule Associated Protein Tau Dysregulates its Cellular Localization. J. Alzheimers Dis..

[126] Cente M., Filipcik P., Mandakova S., Zilka N., Krajciova G., Novak M. (2009). Expression of a Truncated Human Tau Protein Induces Aqueous-Phase Free Radicals in a Rat Model of Tauopathy: Implications for Targeted Antioxidative Therapy. J. Alzheimers Dis..

[127] Su X.-Y., Wu W.-H., Huang Z.-P., Hu J., Lei P., Yu C.-H., Zhao Y.-F., Li Y.-M. (2007). Hydrogen peroxide can be generated by tau in the presence of Cu(II). Biochem. Biophys. Res. Commun..

[128] Cheng Y., Bai F. (2018). The Association of Tau With Mitochondrial Dysfunction in Alzheimer’s Disease. Front. Neurosci..

[129] Prentice H., Modi J.P., Wu J.-Y. (2015). Mechanisms of Neuronal Protection against Excitotoxicity, Endoplasmic Reticulum Stress, and Mitochondrial Dysfunction in Stroke and Neurodegenerative Diseases. Oxidative Med. Cell. Longev..

[130] Teixeira J., Silva T., Andrade P.B., Borges F. (2013). Alzheimer’s Disease and Antioxidant Therapy: How Long How Far?. Curr. Med. Chem..

[131] Novak V., Rogelj B., Župunski V. (2021). Therapeutic Potential of Polyphenols in Amyotrophic Lateral Sclerosis and Frontotemporal Dementia. Antioxidants.

[132] Gonzalez-Zulueta M., Ensz L.M., Mukhina G., Lebovitz R.M., Zwacka R.M., Engelhardt J.F., Oberley L.W., Dawson V.L., Dawson T.M. (1998). Manganese Superoxide Dismutase Protects nNOS Neurons from NMDA and Nitric Oxide-Mediated Neurotoxicity. J. Neurosci..

[133] Nguyen T., Huang H.C., Pickett C.B. (2000). Transcriptional Regulation of the Antioxidant Response Element. Activation by Nrf2 and Repression by MafK. J. Biol. Chem..

[134] Jaiswal A.K. (2004). Nrf2 signaling in coordinated activation of antioxidant gene expression. Free Radic. Biol. Med..

[135] Tapias V., Jainuddin S., Ahuja M., Stack C., Elipenahli C., Vignisse J., Gerges M., Starkova N., Xu H., Starkov A.A. (2018). Benfotiamine treatment activates the Nrf2/ARE pathway and is neuroprotective in a transgenic mouse model of tauopathy. Hum. Mol. Genet..

[136] Stack C., Jainuddin S., Elipenahli C., Gerges M., Starkova N., Starkov A.A., Jové M., Portero-Otin M., Launay N., Pujol A. (2014). Methylene blue upregulates Nrf2/ARE genes and prevents tau-related neurotoxicity. Hum. Mol. Genet..

[137] Cuadrado A., Kügler S., Lastres-Becker I. (2018). Pharmacological targeting of GSK-3 and NRF2 provides neuroprotection in a preclinical model of tauopathy. Redox Biol..

[138] Bahn G., Jo D.-G. (2019). Therapeutic Approaches to Alzheimer’s Disease through Modulation of NRF2. Neuromol. Mol. Med..

[139] Nishida S. (2005). Metabolic Effects of Melatonin on Oxidative Stress and Diabetes Mellitus. Endocrine.

[140] Tan D.-X., Reiter R.J., Manchester L.C., Yan M.-T., El-Sawi M., Sainz R.M., Mayo J.C., Kohen R., Allegra M., Hardelan R. (2002). Chemical and Physical Properties and Potential Mechanisms: Melatonin as a Broad Spectrum Antioxidant and Free Radical Scavenger. Curr. Top. Med. Chem..

[141] Zhang H.-M., Zhang Y. (2014). Melatonin: A well-documented antioxidant with conditional pro-oxidant actions. J. Pineal Res..

[142] Tan D.-X., Manchester L.C., Terron M.P., Flores L.J., Reiter R.J. (2007). One molecule, many derivatives: A never-ending interaction of melatonin with reactive oxygen and nitrogen species?. J. Pineal Res..

[143] Reiter R.J., Tan D.-X., Manchester L.C., Qi W. (2001). Biochemical Reactivity of Melatonin with Reactive Oxygen and Nitrogen Species: A Review of the Evidence. Cell Biochem. Biophys..

[144] Galano A., Tan D.X., Reiter R.J. (2011). Melatonin as a natural ally against oxidative stress: A physicochemical examination. J. Pineal Res..

[145] Galano A., Tan D.X., Reiter R.J. (2013). On the free radical scavenging activities of melatonin’s metabolites, AFMK and AMK. J. Pineal Res..

[146] Pozo D., Reiter R.J., Calvo J.R., Guerrero J.M. (1994). Physiological concentrations of melatonin inhibit nitric oxide synthase in rat cerebellum. Life Sci..

[147] Pozo D., Reiter R.J., Calvo J.R., Guerrero J.M. (1997). Inhibition of cerebellar nitric oxide synthase and cyclic GMP production by melatonin via complex formation with calmodulin. J. Cell. Biochem..

[148] Das R., Balmik A.A., Chinnathambi S. (2020). Melatonin Reduces GSK3β-Mediated Tau Phosphorylation, Enhances Nrf2 Nuclear Translocation and Anti-Inflammation. ASN Neuro.

[149] Li X.-C., Wang Z.-F., Zhang J.-X., Wang Q., Wang J.-Z. (2005). Effect of melatonin on calyculin A-induced tau hyperphosphorylation. Eur. J. Pharmacol..

[150] Deng Y.-Q., Xu G., Duan P., Zhang Q., Wang J.-Z. (2005). Effects of melatonin on wortmannin-induced tau hyperphosphorylation. Acta Pharmacol. Sin..

[151] Wang D.-L., Ling Z.-Q., Cao F.-Y., Zhu L.-Q., Wang J.-Z. (2004). Melatonin attenuates isoproterenol-induced protein kinase A overactivation and tau hyperphosphorylation in rat brain. J. Pineal Res..

[152] Sies H., Stahl W. (1995). Vitamins E and C, beta-carotene, and other carotenoids as antioxidants. Am. J. Clin. Nutr..

[153] Nakashima H., Ishihara T., Yokota O., Terada S., Trojanowski J.Q., Lee V.M.-Y., Kuroda S. (2004). Effects of α-tocopherol on an animal model of tauopathies. Free Radic. Biol. Med..

[154] Devore E.E., Grodstein F., Van Rooij F.J., Hofman A., Stampfer M.J., Witteman J.C., Breteler M.M. (2010). Dietary Antioxidants and Long-term Risk of Dementia. Arch. Neurol..

[155] Sung S., Yao Y., Uryu K., Yang H., Lee V.M., Trojanowski J.Q., Praticò D. (2003). Early Vitamin E supplementation in young but not aged mice reduces Aβ levels and amyloid deposition in a transgenic model of Alzheimer’s disease. FASEB J..

[156] Sano M., Ernesto C., Thomas R.G., Klauber M.R., Schafer K., Grundman M., Woodbury P., Growdon J., Cotman C.W., Pfeiffer E. (1997). A Controlled Trial of Selegiline, Alpha-Tocopherol, or Both as Treatment for Alzheimer’s Disease. N. Engl. J. Med..

[157] Pavlik V.N., Doody R.S., Rountree S.D., Darby E.J. (2010). Vitamin E Use Is Associated with Improved Survival in an Alzheimer’s Disease Cohort. Dement. Geriatr. Cogn. Disord..

[158] Jiang T., Sun Q., Chen S. (2016). Oxidative stress: A major pathogenesis and potential therapeutic target of antioxidative agents in Parkinson’s disease and Alzheimer’s disease. Prog. Neurobiol..

[159] Lloret A., Badía M.-C., Mora N.J., Pallardó F.V., Alonso M.-D., Viña J. (2009). Vitamin E Paradox in Alzheimer’s Disease: It Does Not Prevent Loss of Cognition and May Even Be Detrimental. J. Alzheimers Dis..

[160] Grosso G., Bei R., Mistretta A., Marventano S., Calabrese G., Masuelli L., Giganti M.G., Modesti A., Galvano F., Gazzolo D. (2013). Effects of Vitamin C on Health: A Review of Evidence. Front. Biosci..

[161] Duarte T.L., Lunec J. (2005). ReviewPart of the Series: From Dietary Antioxidants to Regulators in Cellular Signalling and Gene ExpressionReview: When Is an Antioxidant Not an Antioxidant? A Review of Novel Actions and Reactions of Vitamin C. Free. Radic. Res..

[162] Harrison F.E. (2012). A Critical Review of Vitamin C for the Prevention of Age-Related Cognitive Decline and Alzheimer’s Disease. J. Alzheimers Dis..

[163] Monacelli F., Acquarone E., Giannotti C., Borghi R., Nencioni A. (2017). Vitamin C, Aging and Alzheimer’s Disease. Nutrients.

[164] Kook S.-Y., Lee K.-M., Kim Y., Cha M.-Y., Kang S., Baik S.H., Lee H., Park R., Mook-Jung I. (2014). High-dose of vitamin C supplementation reduces amyloid plaque burden and ameliorates pathological changes in the brain of 5XFAD mice. Cell Death Dis..

[165] Heo J.-H., Lee H., Lee K.-M. (2013). The Possible Role of Antioxidant Vitamin C in Alzheimer’s Disease Treatment and Prevention. Am. J. Alzheimers Dis. Other Dement..

[166] Murakami K., Murata N., Ozawa Y., Kinoshita N., Irie K., Shirasawa T., Shimizu T. (2011). Vitamin C Restores Behavioral Deficits and Amyloid-β Oligomerization without Affecting Plaque Formation in a Mouse Model of Alzheimer’s Disease. J. Alzheimers Dis..

[167] Arlt S., Müller-Thomsen T., Beisiegel U., Kontush A. (2012). Effect of One-Year Vitamin C- and E-Supplementation on Cerebrospinal Fluid Oxidation Parameters and Clinical Course in Alzheimer’s Disease. Neurochem. Res..

[168] Galasko D.R., Peskind E., Clark C.M., Quinn J.F., Ringman J.M., Jicha G.A., Cotman C., Cottrell B., Montine T.J., Thomas R.G. (2012). Antioxidants for Alzheimer Disease. Arch. Neurol..

[169] Upritchard J.E., Schuurman C.R., Wiersma A., Tijburg L.B., Coolen S.A., Rijken P.J., Wiseman S.A. (2003). Spread supplemented with moderate doses of vitamin E and carotenoids reduces lipid peroxidation in healthy, nonsmoking adults. Am. J. Clin. Nutr..

[170] Sandhir R., Mehrotra A., Kamboj S.S. (2010). Lycopene prevents 3-nitropropionic acid-induced mitochondrial oxidative stress and dysfunctions in nervous system. Neurochem. Int..

[171] Sachdeva A.K., Chopra K. (2015). Lycopene abrogates Aβ(1–42)-mediated neuroinflammatory cascade in an experimental model of Alzheimer’s disease. J. Nutr. Biochem..

[172] Qu M., Jiang Z., Liao Y., Song Z., Nan X. (2016). Lycopene Prevents Amyloid [Beta]-Induced Mitochondrial Oxidative Stress and Dysfunctions in Cultured Rat Cortical Neurons. Neurochem. Res..

[173] de Oliveira B.F., Veloso C.A., Nogueira-Machado J.A., de Moraes E.N., dos Santos R.R., Cintra M.T.G., Chaves M.M. (2012). Ascorbic acid, alpha-tocopherol, and beta-carotene reduce oxidative stress and proinflammatory cytokines in mononuclear cells of Alzheimer’s disease patients. Nutr. Neurosci..

[174] Kamat C.D., Gadal S., Mhatre M., Williamson K.S., Pye Q.N., Hensley K. (2008). Antioxidants in Central Nervous System Diseases: Preclinical Promise and Translational Challenges. J. Alzheimers Dis..

[175] Vivarelli F., Canistro D., Cirillo S., Papi A., Spisni E., Vornoli A., Croce C.M.D., Longo V., Franchi P., Filippi S. (2019). Co-carcinogenic effects of vitamin E in prostate. Sci. Rep..

[176] Miller E.R., Pastor-Barriuso R., Dalal D., Riemersma R.A., Appel L.J., Guallar E. (2005). Meta-Analysis: High-Dosage Vitamin E Supplementation May Increase All-Cause Mortality. Ann. Intern. Med..

[177] Bjelakovic G., Nikolova D., Gluud L.L., Simonetti R.G., Gluud C. (2007). Mortality in Randomized Trials of Antioxidant Supplements for Primary and Secondary Prevention: Systematic review and meta-analysis. JAMA.

[178] Ross J.A., Kasum C.M. (2002). DIETARY FLAVONOIDS: Bioavailability, Metabolic Effects, and Safety. Annu. Rev. Nutr..

[179] Swallah M.S., Sun H., Affoh R., Fu H., Yu H. (2020). Antioxidant Potential Overviews of Secondary Metabolites (Polyphenols) in Fruits. Int. J. Food Sci..

[180] Sun X.-Y., Dong Q.-X., Zhu J., Sun X., Zhang L.-F., Qiu M., Yu X.-L., Liu R.-T. (2019). Resveratrol Rescues Tau-Induced Cognitive Deficits and Neuropathology in a Mouse Model of Tauopathy. Curr. Alzheimer Res..

[181] Ashrafizadeh M., Zarrabi A., Najafi M., Samarghandian S., Mohammadinejad R., Ahn K.S. (2020). Resveratrol targeting tau proteins, amyloid-beta aggregations, and their adverse effects: An updated review. Phytother. Res..

[182] Cosín-Tomàs M., Senserrich J., Arumí-Planas M., Alquézar C., Pallàs M., Martín-Requero Á., Suñol C., Kaliman P., Sanfeliu C. (2019). Role of Resveratrol and Selenium on Oxidative Stress and Expression of Antioxidant and Anti-Aging Genes in Immortalized Lymphocytes from Alzheimer’s Disease Patients. Nutrients.

[183] Griñán-Ferré C., Bellver-Sanchis A., Izquierdo V., Corpas R., Roig-Soriano J., Chillón M., Andres-Lacueva C., Somogyvári M., Sőti C., Sanfeliu C. (2021). The pleiotropic neuroprotective effects of resveratrol in cognitive decline and Alzheimer’s disease pathology: From antioxidant to epigenetic therapy. Ageing Res. Rev..

[184] Sarroca S., Gatius A., Rodríguez-Farré E., Vilchez D., Pallàs M., Griñán-Ferré C., Sanfeliu C., Corpas R. (2021). Resveratrol confers neuroprotection against high-fat diet in a mouse model of Alzheimer’s disease via modulation of proteolytic mechanisms. J. Nutr. Biochem..

[185] Tian B., Liu J. (2020). Resveratrol: A review of plant sources, synthesis, stability, modification and food application. J. Sci. Food Agric..

[186] Tellone E., Galtieri A., Russo A., Giardina B., Ficarra S. (2015). Resveratrol: A Focus on Several Neurodegenerative Diseases. Oxidative Med. Cell. Longev..

[187] Subbaramaiah K., Chung W.J., Michaluart P., Telang N., Tanabe T., Inoue H., Jang M., Pezzuto J.M., Dannenberg A.J. (1998). Resveratrol Inhibits Cyclooxygenase-2 Transcription and Activity in Phorbol Ester-treated Human Mammary Epithelial Cells. J. Biol. Chem..

[188] Fontecave M., Lepoivre M., Elleingand E., Gerez C., Guittet O. (1998). Resveratrol, a remarkable inhibitor of ribonucleotide reductase. FEBS Lett..

[189] Stewart J.R., Ward N.E., Ioannides C.G., O’Brian C.A. (1999). Resveratrol Preferentially Inhibits Protein Kinase C-Catalyzed Phosphorylation of a Cofactor-Independent, Arginine-Rich Protein Substrate by a Novel Mechanism. Biochemistry.

[190] Locatelli G.A., Savio M., Forti L., Shevelev I., Ramadan K., Stivala L.A., Vannini V., Hübscher U., Spadari S., Maga G. (2005). Inhibition of mammalian DNA polymerases by resveratrol: Mechanism and structural determinants. Biochem. J..

[191] Basly J.-P., Marre-Fournier F., Le Bail J.-C., Habrioux G., Chulia A.J. (2000). Estrogenic/antiestrogenic and scavenging properties of (E)- and (Z)-resveratrol. Life Sci..

[192] Marumo M., Ekawa K., Wakabayashi I. (2020). Resveratrol inhibits Ca^2+^ signals and aggregation of platelets. Environ. Health Prev. Med..

[193] Lagouge M., Argmann C., Gerhart-Hines Z., Meziane H., Lerin C., Daussin F., Messadeq N., Milne J., Lambert P., Elliott P. (2006). Resveratrol improves mitochondrial function and protects against metabolic disease by activating SIRT1 and PGC-1α. Cell.

[194] Shang J., Chen L., Xiao F., Sun H., Ding H., Xiao H. (2008). Resveratrol improves non-alcoholic fatty liver disease by activating AMP-activated protein kinase. Acta Pharmacol. Sin..

[195] Cao D., Wang M., Qiu X., Liu D., Jiang H., Yang N., Xu R.-M. (2015). Structural basis for allosteric, substrate-dependent stimulation of SIRT1 activity by resveratrol. Genes Dev..

[196] Min S.-W., Cho S.-H., Zhou Y., Schroeder S., Haroutunian V., Seeley W.W., Huang E.J., Shen Y., Masliah E., Mukherjee C. (2010). Acetylation of Tau Inhibits Its Degradation and Contributes to Tauopathy. Neuron.

[197] Cook C., Stankowski J.N., Carlomagno Y., Stetler C., Petrucelli L. (2014). Acetylation: A new key to unlock tau’s role in neurodegeneration. Alzheimer’s Res. Ther..

[198] Min S.-W., Chen X., Tracy T.E., Li Y., Zhou Y., Wang C., Shirakawa K., Minami S.S., Defensor E., Mok S.-A. (2015). Critical role of acetylation in tau-mediated neurodegeneration and cognitive deficits. Nat. Med..

[199] Irwin D., Cohen T.J., Grossman M., Arnold S.E., McCarty-Wood E., Van Deerlin V.M., Lee V.M.-Y., Trojanowski J.Q. (2013). Acetylated Tau Neuropathology in Sporadic and Hereditary Tauopathies. Am. J. Pathol..

[200] Min S.-W., Sohn P.D., Li Y., Devidze N., Johnson J.R., Krogan N.J., Masliah E., Mok S.-A., Gestwicki J.E., Gan L. (2018). SIRT1 Deacetylates Tau and Reduces Pathogenic Tau Spread in a Mouse Model of Tauopathy. J. Neurosci..

[201] Schweiger S., Matthes F., Posey K., Kickstein E., Weber S., Hettich M.M., Pfurtscheller S., Ehninger D., Schneider R., Krauß S. (2017). Resveratrol induces dephosphorylation of Tau by interfering with the MID1-PP2A complex. Sci. Rep..

[202] Patel K.R., Scott E., Brown V.A., Gescher A.J., Steward W.P., Brown K. (2011). Clinical trials of resveratrol. Ann. N. Y. Acad. Sci..

[203] Turner R.S., Thomas R.G., Craft S., Van Dyck C.H., Mintzer J., Reynolds B.A., Brewer J.B., Rissman R.A., Raman R., Aisen P.S. (2015). A randomized, double-blind, placebo-controlled trial of resveratrol for Alzheimer disease. Neurology.

[204] Moussa C., Hebron M., Huang X., Ahn J., Rissman R.A., Aisen P.S., Turner R.S. (2017). Resveratrol regulates neuro-inflammation and induces adaptive immunity in Alzheimer’s disease. J. Neuroinflam..

[205] Sawda C., Moussa C., Turner R.S. (2017). Resveratrol for Alzheimer’s Disease. Ann. N. Y. Acad. Sci..

[206] Hu S., Maiti P., Ma Q., Zuo X., Jones M.R., Cole G.M., Frautschy S.A. (2015). Clinical development of curcumin in neurodegenerative disease. Expert Rev. Neurother..

[207] Sivanantharajah L., Mudher A. (2022). Curcumin as a Holistic Treatment for Tau Pathology. Front. Pharmacol..

[208] Rane J.S., Bhaumik P., Panda D. (2017). Curcumin Inhibits Tau Aggregation and Disintegrates Preformed Tau Filaments in vitro. J. Alzheimer’s Dis..

[209] Cascio F.L., Puangmalai N., Ellsworth A., Bucchieri F., Pace A., Piccionello A.P., Kayed R. (2019). Toxic Tau Oligomers Modulated by Novel Curcumin Derivatives. Sci. Rep..

[210] Den Haan J., Morrema T.H.J., Rozemuller A.J., Bouwman F.H., Hoozemans J.J.M. (2018). Different curcumin forms selectively bind fibrillar amyloid beta in post mortem Alzheimer’s disease brains: Implications for in-vivo diagnostics. Acta Neuropathol. Commun..

[211] Miyasaka T., Xie C., Yoshimura S., Shinzaki Y., Yoshina S., Kage-Nakadai E., Mitani S., Ihara Y. (2016). Curcumin improves tau-induced neuronal dysfunction of nematodes. Neurobiol. Aging.

[212] Hagl S., Kocher A., Schiborr C., Kolesova N., Frank J., Eckert G.P. (2015). Curcumin micelles improve mitochondrial function in neuronal PC12 cells and brains of NMRI mice—Impact on bioavailability. Neurochem. Int..

[213] Sundaram J.R., Poore C.P., Sulaimee N.H.B., Pareek T., Cheong W.F., Wenk M.R., Pant H.C., Frautschy S.A., Low C.-M., Kesavapany S. (2017). Curcumin Ameliorates Neuroinflammation, Neurodegeneration, and Memory Deficits in p25 Transgenic Mouse Model that Bears Hallmarks of Alzheimer’s Disease. J. Alzheimers Dis..

[214] Ma Z., Wang N., He H., Tang X. (2019). Pharmaceutical strategies of improving oral systemic bioavailability of curcumin for clinical application. J. Control. Release.

[215] Voulgaropoulou S.D., Van Amelsvoort T.A.M.J., Prickaerts J., Vingerhoets C. (2019). The effect of curcumin on cognition in Alzheimer’s disease and healthy aging: A systematic review of pre-clinical and clinical studies. Brain Res..

[216] Scalbert A., Morand C., Manach C., Rémésy C. (2002). Absorption and metabolism of polyphenols in the gut and impact on health. Biomed. Pharmacother..

[217] Velásquez-Jiménez D., Corella-Salazar D.A., Zuñiga-Martínez B.S., Domínguez-Avila J.A., Montiel-Herrera M., Salazar-López N.J., Rodrigo-Garcia J., Villegas-Ochoa M.A., González-Aguilar G.A. (2021). Phenolic compounds that cross the blood–brain barrier exert positive health effects as central nervous system antioxidants. Food Funct..

[218] Rezaee N., Fernando W.B., Hone E., Sohrabi H.R., Johnson S.K., Gunzburg S., Martins R.N. (2021). Potential of Sorghum Polyphenols to Prevent and Treat Alzheimer’s Disease: A Review Article. Front. Aging Neurosci..

[219] Szwajgier D., Borowiec K., Pustelniak K. (2017). The Neuroprotective Effects of Phenolic Acids: Molecular Mechanism of Action. Nutrients.

[220] Xu Z., Chen S., Li X., Luo G., Li L., Le W. (2006). Neuroprotective Effects of (-)-Epigallocatechin-3-gallate in a Transgenic Mouse Model of Amyotrophic Lateral Sclerosis. Neurochem. Res..

[221] Che F., Wang G., Yu J., Wang X., Lu Y., Fu Q., Su Q., Jiang J., Du Y. (2017). Effects of epigallocatechin-3-gallate on iron metabolism in spinal cord motor neurons. Mol. Med. Rep..

[222] Srinivasan E., Rajasekaran R. (2017). Probing the inhibitory activity of epigallocatechin-gallate on toxic aggregates of mutant (L84F) SOD1 protein through geometry based sampling and steered molecular dynamics. J. Mol. Graph. Model..

[223] Taniguchi S., Suzuki N., Masuda M., Hisanaga S.-I., Iwatsubo T., Goedert M., Hasegawa M. (2005). Inhibition of Heparin-induced Tau Filament Formation by Phenothiazines, Polyphenols, and Porphyrins. J. Biol. Chem..

[224] Koh S.H., Kim S.H., Kwon H., Kim J.G., Kim J.H., Yang K.-H., Yu H.-J., Do B.R., Kim K.S., Jung H.K. (2004). Phosphatidylinositol-3 Kinase/Akt and GSK-3 Mediated Cytoprotective Effect of Epigallocatechin Gallate on Oxidative Stress-Injured Neuronal-Differentiated N18D3 Cells. NeuroToxicology.

[225] Cho J.H., Johnson G.V.W. (2003). Glycogen Synthase Kinase 3β Phosphorylates Tau at Both Primed and Unprimed Sites: Differential Impact on Microtubule Binding. J. Biol. Chem..

[226] Wang H., Wang H., Cheng H., Che Z. (2016). Ameliorating effect of luteolin on memory impairment in an Alzheimer’s disease model. Mol. Med. Rep..

[227] Ali F., Rahul, Jyoti S., Naz F., Ashafaq M., Shahid M., Siddique Y.H. (2018). Therapeutic potential of luteolin in transgenic Drosophila model of Alzheimer’s disease. Neurosci. Lett..

[228] Assogna M., Casula E.P., Borghi I., Bonnì S., Samà D., Motta C., Di Lorenzo F., D’Acunto A., Porrazzini F., Minei M. (2020). Effects of Palmitoylethanolamide Combined with Luteoline on Frontal Lobe Functions, High Frequency Oscillations, and GABAergic Transmission in Patients with Frontotemporal Dementia. J. Alzheimer’s Dis..

[229] Collins S.M., Surette M., Bercik P. (2012). The interplay between the intestinal microbiota and the brain. Nat. Rev. Microbiol..

[230] Bhattacharjee S., Lukiw W. (2013). Alzheimer’s Disease and the Microbiome. Front. Cell. Neurosci..

[231] Kowalski K., Mulak A. (2019). Brain-Gut-Microbiota Axis in Alzheimer’s Disease. J. Neurogastroenterol. Motil..

[232] Friedland R.P., Chapman M.R. (2017). The role of microbial amyloid in neurodegeneration. PLOS Pathog..

[233] Jones R.M., Mercante J.W., Neish A.S. (2012). Reactive Oxygen Production Induced by the Gut Microbiota: Pharmacotherapeutic Implications. Curr. Med. Chem..

[234] Cobley J.N., Fiorello M.L., Bailey D.M. (2018). 13 reasons why the brain is susceptible to oxidative stress. Redox Biol..

[235] Siedlak S.L., Casadesus G., Webber K.M., Pappolla M.A., Atwood C.S., Smith M.A., Perry G. (2009). Chronic antioxidant therapy reduces oxidative stress in a mouse model of Alzheimer’s disease. Free Radic. Res..

[236] Zhang Y.-H., Wang D.-W., Xu S.-F., Zhang S., Fan Y.-G., Yang Y.-Y., Guo S.-Q., Wang S., Guo T., Wang Z.-Y. (2017). α-Lipoic acid improves abnormal behavior by mitigation of oxidative stress, inflammation, ferroptosis, and tauopathy in P301S Tau transgenic mice. Redox Biol..

[237] Zarini-Gakiye E., Vaezi G., Parivar K., Sanadgol N. (2021). Age and Dose-Dependent Effects of Alpha-Lipoic Acid on Human Microtubule- Associated Protein Tau-Induced Endoplasmic Reticulum Unfolded Protein Response: Implications for Alzheimer’s Disease. CNS Neurol. Disord. Drug Targets.

[238] Quinn J., Bussiere J.R., Hammond R.S., Montine T.J., Henson E., Jones R.E., Stackman R.W. (2007). Chronic dietary α-lipoic acid reduces deficits in hippocampal memory of aged Tg2576 mice. Neurobiol. Aging.

[239] Bonakdar R.A., Guarneri E. (2005). Coenzyme Q10. Am. Fam. Physician.

[240] Dumont M., Kipiani K., Yu F., Wille E., Katz M., Calingasan N.Y., Gouras G.K., Lin M.T., Beal M.F. (2011). Coenzyme Q10 Decreases Amyloid Pathology and Improves Behavior in a Transgenic Mouse Model of Alzheimer’s Disease. J. Alzheimers Dis..

[241] Elipenahli C., Stack C., Jainuddin S., Gerges M., Yang L., Starkov A., Beal M.F., Dumont M. (2012). Behavioral Improvement after Chronic Administration of Coenzyme Q10 in P301S Transgenic Mice. J. Alzheimers Dis..

[242] Yang M., Lian N., Yu Y., Wang Y., Xie K., Yu Y. (2020). Coenzyme Q10 alleviates sevoflurane-induced neuroinflammation by regulating the levels of apolipoprotein E and phosphorylated tau protein in mouse hippocampal neurons. Mol. Med. Rep..

[243] Ferrante K.L., Shefner J., Zhang H., Betensky R., O’Brien M., Yu H., Fantasia M., Taft J., Beal M.F., Traynor B. (2005). Tolerance of high-dose (3,000 mg/day) coenzyme Q10 in ALS. Neurology.

[244] Stamelou M., Reuss A., Pilatus U., Magerkurth J., Niklowitz P., Eggert K.M., Krisp A., Menke T., Schade-Brittinger C., Oertel W.H. (2008). Short-term effects of coenzyme Q_10_ in progressive supranuclear palsy: A randomized, placebo-controlled trial. Mov. Disord..

[245] Santa-Marıa I., Santpere G., MacDonald M.J., de Barreda E.G., Hernandez F., Moreno F.J., Ferrer I., Avila J. (2008). Coenzyme Q Induces Tau Aggregation, Tau Filaments, and Hirano Bodies. J. Neuropathol. Exp. Neurol..

[246] Kelso G.F., Porteous C.M., Coulter C.V., Hughes G., Porteous W.K., Ledgerwood E.C., Smith R.A., Murphy M.P. (2001). Selective Targeting of a Redox-active Ubiquinone to Mitochondria within Cells: Antioxidant and antiapoptotic properties. J. Biol. Chem..

[247] Young M.L., Franklin J.L. (2019). The mitochondria-targeted antioxidant MitoQ inhibits memory loss, neuropathology, and extends lifespan in aged 3xTg-AD mice. Mol. Cell. Neurosci..

[248] Esteras N., Kopach O., Maiolino M., Lariccia V., Amoroso S., Qamar S., Wray S., Rusakov D.A., Jaganjac M., Abramov A.Y. (2022). Mitochondrial ROS control neuronal excitability and cell fate in frontotemporal dementia. Alzheimers Dement..

[249] Abourashed E.A. (2013). Bioavailability of Plant-Derived Antioxidants. Antioxidants.

[250] Vaiserman A., Koliada A., Zayachkivska A., Lushchak O. (2020). Nanodelivery of Natural Antioxidants: An Anti-aging Perspective. Front. Bioeng. Biotechnol..

[251] Hamishehkar H., Ranjdoost F., Asgharian P., Mahmoodpoor A., Sanaie S. (2016). Vitamins, Are They Safe?. Adv. Pharm. Bull..

[252] Liu Z., Zhou T., Ziegler A.C., Dimitrion P., Zuo L. (2017). Oxidative Stress in Neurodegenerative Diseases: From Molecular Mechanisms to Clinical Applications. Oxidative Med. Cell. Longev..

[253] Gilgun-Sherki Y., Melamed E., Offen D. (2001). Oxidative stress induced-neurodegenerative diseases: The need for antioxidants that penetrate the blood brain barrier. Neuropharmacology.

[254] Pinto M., Benfeito S., Fernandes C., Borges F., Martin C.R., Preedy V.R. (2020). Chapter 9—Antioxidant Therapy, Oxidative Stress, and Blood-Brain Barrier: The Road of Dietary Antioxidants. Oxidative Stress and Dietary Antioxidants in Neurological Diseases.

[255] Dawson T.M., Golde T.E., Lagier-Tourenne C. (2018). Animal models of neurodegenerative diseases. Nat. Neurosci..

[256] Han S.S., Williams L.A., Eggan K.C. (2011). Constructing and Deconstructing Stem Cell Models of Neurological Disease. Neuron.

[257] Logan S., Arzua T., Canfield S.G., Seminary E.R., Sison S.L., Ebert A.D., Bai X. (2019). Studying Human Neurological Disorders Using Induced Pluripotent Stem Cells: From 2D Monolayer to 3D Organoid and Blood Brain Barrier Models. Compr. Physiol..

